# Therapeutic Ultrasound for Multimodal Cancer Treatment: A Spotlight on Breast Cancer

**DOI:** 10.1146/annurev-bioeng-103023-111151

**Published:** 2025-02-19

**Authors:** Zehra E.F. Demir, Natasha D. Sheybani

**Affiliations:** 1 Department of Biomedical Engineering, University of Virginia, Charlottesville, Virginia, USA; 2 Department of Radiology and Medical Imaging, University of Virginia, Charlottesville, Virginia, USA

**Keywords:** focused ultrasound, breast cancer, metastasis, cancer immunotherapy, image-guided therapy, HIFU, drug delivery

## Abstract

Cancer remains a leading cause of mortality worldwide, and the demand for improved efficacy, precision, and safety of management options has never been greater. Focused ultrasound (FUS) is a rapidly emerging strategy for nonionizing, noninvasive intervention that holds promise for the multimodal treatment of solid cancers. Owing to its versatile array of bioeffects, this technology is now being evaluated across preclinical and clinical oncology trials for tumor ablation, therapeutic delivery, radiosensitization, sonodynamic therapy, and enhancement of tumor-specific immune responses. Given the breadth of this burgeoning domain, this review places a spotlight on recent advancements in breast cancer care to exemplify the multifaceted role of FUS technology for oncology indications—outlining physical principles of FUS-mediated thermal and mechanical bioeffects, giving an overview of results from recent preclinical and clinical studies investigating FUS with and without adjunct therapeutics in primary or disseminated breast cancer settings, and offering perspectives on the future of the field.

## INTRODUCTION

### Breast Cancer: Global Landscape and Critical Gaps

Breast cancer (BC) remains the most common cancer diagnosis and second-leading cause of cancer-related deaths in women worldwide. The morbidity and mortality associated with BC are frequently attributable to metastases (for instance, to lung, liver, bone, or lymph nodes), with brain metastases being among the most formidable (1). In 2024, the National Cancer Institute estimated 310,780 new breast cancer cases in the United States alone, with its prevalence on the rise. Among these women, those of African American descent are disproportionately affected by BC, with incidence continuing to rise compared with the White population (2).

The diagnosis and prognostication of BCs are rooted in the interlacing of traditional clinico-pathological variables (i.e., tumor size, grade, lymph node positivity, age) with immunohistological benchmarks for proliferation index (Ki67), hormone receptor status [i.e., expression of estrogen receptor (ER) and progesterone receptor (PR)], and human epidermal growth factor receptor (HER2) levels. A summary of BC subtypes is provided in Supplemental Table 1. Briefly, luminal A tumors are characterized by the expression of ER and/or PR, as well as the absence of HER2 and low Ki67 index. These tumors tend to be lower grade, which is associated with higher patient survival rates. Hormone therapy is often the standard of care for this BC subtype (3). Luminal B tumors, which are classified as ER+ and can be PR+, are associated with higher grade, increased Ki67 signature, and a less favorable prognosis. These tumors can be either HER2+ or HER2−, with HER2 expression indicating poorer prognosis compared with luminal A. The overexpression of HER2, in the absence of other hormone receptors, characterizes another tumor subtype that is more aggressive than either the luminal A or B subtypes. Similar to luminal B tumors, HER2+ tumors also tend to have a higher Ki67 index. Tumors that are HER2+ often exhibit a high potential for metastasis, and, despite the availability of targeted therapies, their prevalence has been increasing (by ~30%). HER2+ BCs have a worse prognosis compared with luminal tumors, requiring therapeutic strategies that target the HER2/neu protein, such as trastuzumab, pertuzumab, and tyrosine kinase inhibitors—in addition to standard surgical resection and chemotherapy [e.g., doxorubicin (DOX) and cyclophosphamide, docetaxel, and carboplatin] (4-6). Similar to HER2+ tumors, triple-negative BCs (TNBCs) are also known for their propensity to spread to distal organs. TNBCs tend to be aggressive in nature, lacking therapeutic targets due to the absence of hormone receptors and HER2. There are two subsets of TNBC, basal-like and claudin-low, which bode additional markers distinguishing the two groups. Basal-like TNBC expresses basal markers (i.e., cytokeratin-5, −6, −17), while claudin-low TNBC lacks critical cell–cell adhesion molecules (i.e., claudin-3, −4, −7; E-cadherin; and occludin) (7, 8). Adjuvant chemotherapies, including cisplatin, carboplatin, paclitaxel, docetaxel, and gemcitabine, have shown limited success in treating TNBC (9, 10).

**Figure 1:**
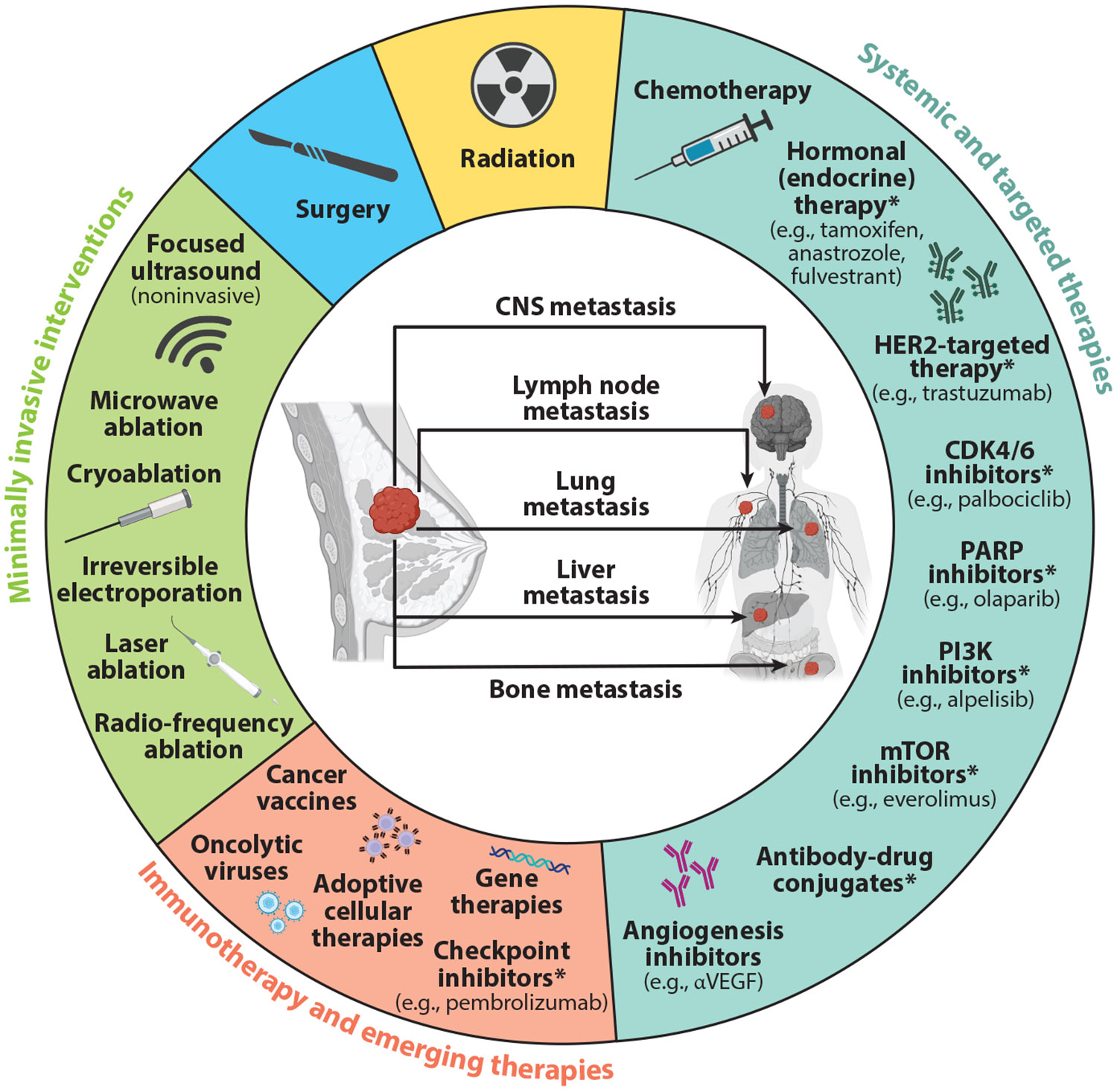
Landscape of breast cancer therapy: overview of conventional and novel treatment modalities for primary or metastatic breast cancers (common sites of metastasis denoted in center of wheel). These therapies include traditional surgery and radiation, systemic and targeted therapies, immunotherapies, emerging treatments, and minimally invasive interventions. Asterisks (*) denote FDA approval of drugs for certain types of breast cancer. Abbreviations: CDK, cyclin-dependent kinase; CNS, central nervous system; FDA, Food and Drug Administration; HER, human epidermal growth factor receptor; mTOR, mammalian target of rapamycin; PARP, poly(ADP-ribose) polymerase; PI3K, phosphoinositide 3-kinase; VEGF, vascular endothelial growth factor. Figure adapted from images created in BioRender; Sheybani N. 2025. https://BioRender.com/o54j510.

The standard of care for BC patients includes surgical resection, chemotherapy, and/or ionizing radiation therapy, with a suite of hormone therapies and other targeted therapies used on a subtype-dependent basis; indeed, these therapies are utilized more commonly in luminal and HER2+ tumors, respectively (1, 11). Outside of conventional interventions, immunotherapies have also emerged as a revolutionary class of drugs leveraging mechanisms of endogenous immune surveillance to combat cancers; unfortunately, solid tumors present unique challenges to the efficacy of immunotherapies, and BCs are no exception to this, with this class of therapies (specifically, checkpoint blockade with pembrolizumab) having to date advanced to Food and Drug Administration (FDA) approval only for specific TNBC settings. Figure 1 provides an overview of the BC therapy landscape.

Collectively, the treatment options for BC carry numerous burdens to the patient. Surgical resection harbors inherent risk as an invasive procedure, often requires extended recovery time, can lead to breast disfigurement and poor cosmesis, and may result in scarring that can bear further unfavorable physiological impacts. Meanwhile, chemotherapy and radiation are notorious for their debilitating short- and long-term off-target toxicities. Immunotherapies, too, commonly face the limitation of immune-related toxicities that range in severity and in some cases even risk lethality (6, 11). With prevalence of BC on the rise and increasingly so in younger populations, there is a clear and urgent need for a quantum leap in both the effectiveness of treatment paradigms and their impact on long-term survivorship—a need shared by myriad cancer types that extend well beyond BC.

In this article, we introduce a rapidly accelerating technology—focused ultrasound (FUS)—that promises to offer unique opportunities for potentiating and/or improving the safety profile of contemporary treatment paradigms in BC—whether they be radiation, chemotherapy, immunotherapy, hormone therapy, targeted therapy, or the like.

### Focused Ultrasound: A Noninvasive, Nontoxic Intervention

FUS is a noninvasive, nonionizing, safe, and repeatable intervention tool. Tracing back to the early 1900s, FUS deploys high-frequency sound waves to precisely target and treat tissue in specific locations within the body, without impact to intervening tissues. The first report of a successful FUS application in cancer (i.e., breast and thyroid cancer) dates back to 1964 (12). Since then, indications and mechanisms of action for FUS technology have expanded exponentially—with oncologic indications being central to this development. To date, approvals of FUS technology have been achieved worldwide across numerous devices and indications—with FUS ablation for benign and malignant breast tumors being among them. While FUS technology is not yet FDA approved for breast tumor applications, steady progress is being made toward this goal through the multitude of clinical efforts geared toward demonstrating its safety, feasibility, and utility.

The completely noninvasive nature of FUS offers the potential to substantially mitigate risks of pain, infection, scarring, or side effects often associated with invasive and even minimally invasive intervention strategies. This is especially important for vulnerable populations—such as the elderly demographic, who are more commonly affected by cancers—as invasive surgeries can increase the risk of complications and can lead to longer recovery times. Furthermore, the repeatability and tunability of FUS offer additional advantages and opportunities for improved precision in the treatment of cancers.

FUS treatments are commonly guided by diagnostic B-mode ultrasound (US) imaging, magnetic resonance imaging (MRI), or, more recently, harmonic motion imaging (HMI) to allow precise targeting, treatment planning, and real-time feedback. As discussed in this review, the therapeutic capabilities of FUS are not limited to tissue ablation but rather span a unique spectrum that enables the same core technology to confer a vast array of thermal and/or mechanical bioeffects. These effects can destroy tumor tissues, amplify DNA damage, enhance therapeutic delivery, promote vasodilation or vascular disruption, invoke immunostimulatory mechanisms, and more (13, 14). Given the vast breadth and depth of the FUS literature, this review places a spotlight on BC to showcase the capabilities of FUS that also extend to the broader oncologic realm. We present a broad overview of physical FUS modalities relevant to BC, provide a comprehensive landscape review of FUS applications in select primary and metastatic BC—with and without adjunct therapeutic approaches—and offer concluding perspectives on the outlook of this burgeoning field.

## PHYSICAL MECHANISMS OF FOCUSED ULTRASOUND AND APPLICATIONS IN BREAST CANCER: AN OVERVIEW

### Hyperthermia

FUS hyperthermia is a technique that uses low-intensity sound waves to effectively “warm” the targeted region or tumor to temperatures within the range of 40–45°C—which, while canonically considered to be sublethal, can confer direct cytotoxicity if applied at prolonged or higher temperature exposures. The downstream impacts of hyperthermia are largely synergistic or sensitizing. These include induction of cellular stresses, which can result in heat shock protein (HSP) expression and promote downstream sterile inflammatory mechanisms; increased blood flow, which can aid drug and nanoparticle delivery; and improved oxygenation, which can sensitize tumor cells to DNA damage such as that conferred by radiotherapy (14-18).

Hyperthermia has also been combined with ultrasound-assisted microbubble (MB) regimens as a novel therapeutic approach in BCs (17, 18). MBs are small, gas-filled bubbles, typically a few microns in diameter, that are used as contrast agents or therapeutic enhancers in US imaging and therapy applications. Typically composed of a gas core surrounded by a stabilizing shell made of lipids, proteins, or polymers, MBs can oscillate and collapse when exposed to FUS waves—in a process known as cavitation. This can enhance the mechanical effects of ultrasound, aiding in the disruption of cell membranes and promoting increased permeability for drug delivery. To this end, numerous BC studies have to date investigated impact to vasculature and blood flow following hyperthermia with MBs, for example, indicating that the duration of hyperthermia therapy impacts the vascular index, wherein longer treatments induce superlative blood flow (17, 18). This combination not only disrupts the vascular system, causing a decrease in oxygen saturation (18), but also damages the tumor and its associated vascular supply (17, 18). In addition to MBs, hyperthermic FUS regimens can also be coadministered with nanoparticles (NPs). One report presented novel tumor-homing/penetrating peptide-functionalized drug-loaded phase-transformation NPs (tLyP-1-10-HCPT-PFP NPs), capable of use for both molecular imaging and precise therapy of tumors with low-intensity FUS exposures. The in vivo results demonstrated effective tumor accumulation of tLyP-1-10-HCPT-PFP NPs, which converted into MBs via acoustic droplet vaporization, improving diagnostic US imaging of tumors. Additional acoustic exposure triggered the release of hydroxycamptothecin (10-HCPT), an analogue of camptothecin and potent antitumor therapeutic agent, leading to a significant reduction in outgrowth of MDA-MB-231 tumors (19). These findings underscore roles for FUS hyperthermia alone and with additional agents such as MBs or NPs—highlighting the depth of potential of combinatory treatment paradigms in this context. Beyond preclinical research, an ongoing clinical trial is currently investigating FUS hyperthermia for the release of lyso-thermosensitive liposomal DOX in combination with cyclophosphamide for metastatic breast cancer treatment [National Clinical Trial (NCT) identifier NCT03749850].

### Thermal Ablation

**Figure 2:**
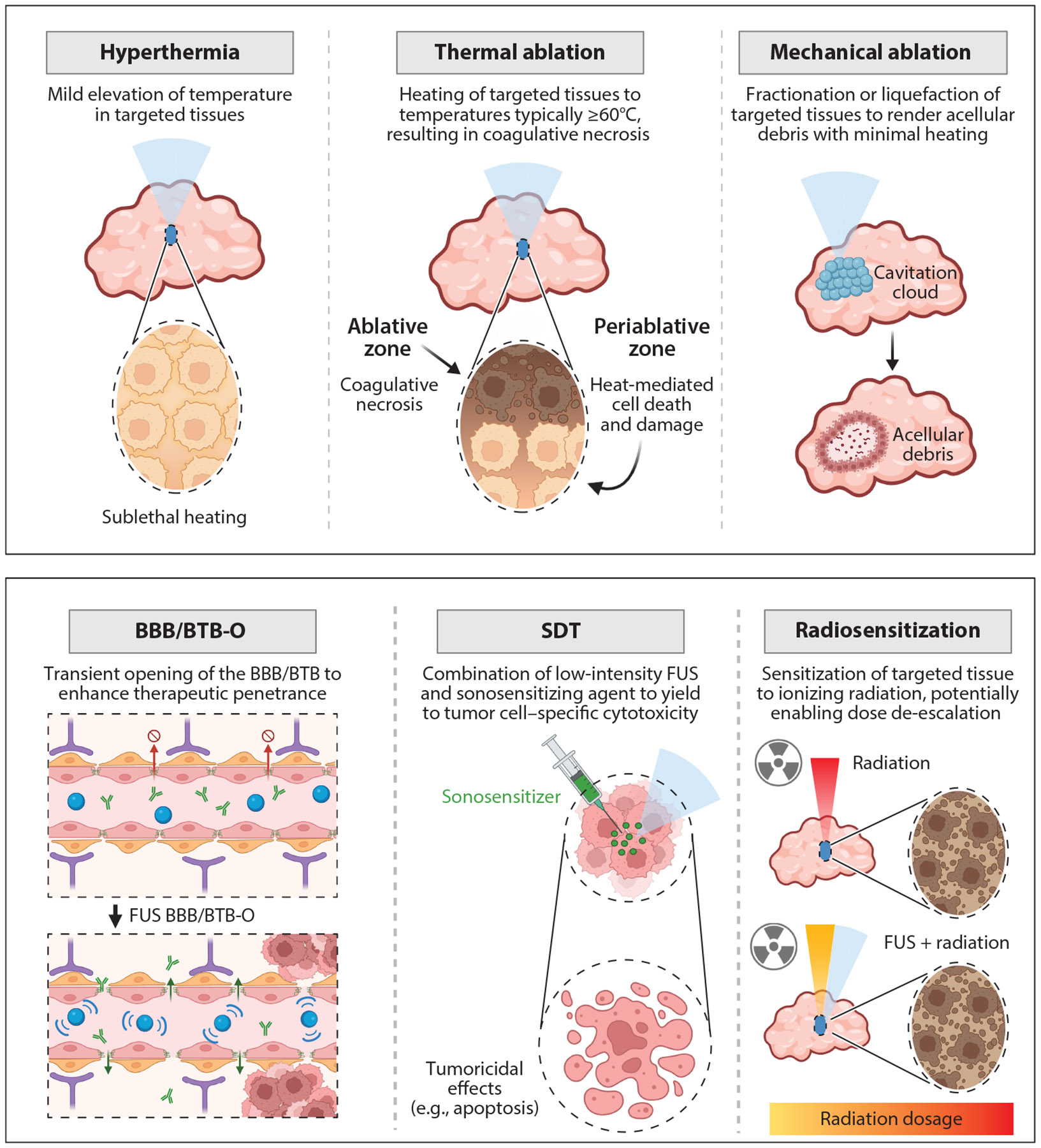
Common FUS modalities in breast cancer: illustrations of FUS mechanisms of action that have been implemented in preclinical and clinical studies of breast cancer to date. The denoted bioeffects span both thermal and mechanical energy deposition and can be modified on the basis of acoustic exposure conditions and/or coadministration with cavitation nuclei (e.g., microbubbles; *blue* in BBB/BTB-O panel), sonosensitizers (*green* in SDT panel), or other agents. Abbreviations: BBB, blood–brain barrier; BBB/BTB-O, BBB/BTB opening; BTB, blood–tumor barrier; FUS, focused ultrasound; SDT, sonodynamic therapy. Figure adapted from images created in BioRender; Sheybani N. 2025. https://BioRender.com/e57w681.

Thermally ablative FUS (T-FUS) operates via application of high-intensity continuous sound waves that can rapidly raise local tissue temperatures to >60°C within the focal zone, while sparing surrounding tissue regions (Figure 2). Concentrated ultrasound beams are briefly maintained at high temperatures within the targeted region, resulting in a volumetric focal zone of precise, instantaneous coagulative necrosis. Within this region of instantaneous tissue destruction, effects such as protein denaturation, irreversible cell growth arrest, and constriction of tumor blood flow can also occur. The focal zone is surrounded by a periablative zone or transitional margin where cells are exposed to thermal dose at tapered levels relative to the immediate focal zone, yielding cellular thermal stresses, upregulation of HSPs, apoptotic events, and other forms of heat-mediated damage to targeted tissues (16, 20-23) (Supplemental Figure 1).

**Figure 3:**
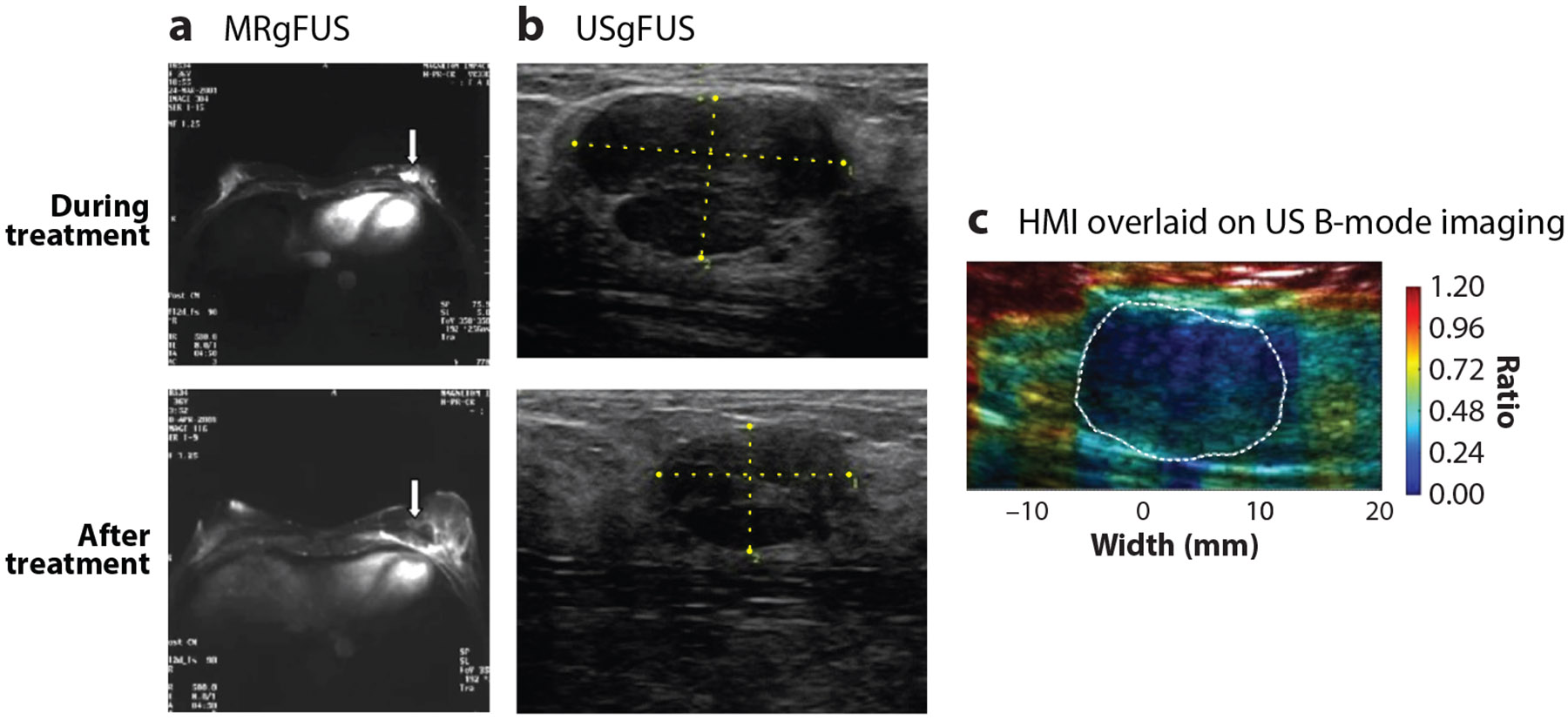
Image-guided FUS for breast tumor lesioning: representative depictions of pre- and posttreatment MRgFUS and USgFUS procedures, as well as representative HMI-guided imaging in BC patients. (*a*) Contrast uptake (*white arrow*) observed during MRgFUS treatment indicates the presence of the tumor, which is no longer visible in the posttreatment image, suggesting coagulative necrosis. (*b*) USgFUS images during and after ablation displaying shrinkage of tumor margins (hypoechoic region denoted by *crosshairs*). (*c*) HMI overlay on B-mode US imaging illustrating the integration of modalities. Tumor borders indicated by white dashed lines. Abbreviations: BC, breast cancer; FUS, focused ultrasound; HMI, harmonic motion imaging; MRgFUS, magnetic resonance–guided FUS; US, ultrasound; USgFUS, ultrasound-guided FUS. Panel *a* adapted with permission from Reference 124. Panel *b* adapted with permission from Reference 23. Panel *c* adapted with permission from Reference 37.

The aforementioned imaging modalities (i.e., US, MRI, and HMI) are uniformly most advanced for guidance of T-FUS procedures and thus discussed here in greater depth (Figure 3). MRI guidance offers the highest spatial resolution among these modalities, as well as robust real-time thermometry capabilities. Several preclinical (24-26) and clinical (27-32) studies have evaluated the impact of MRI-guided T-FUS treatments, most notably in the context of efforts toward increasingly complete ablation of breast lesions. To this end, the strength of MRI guidance is rooted in its demonstrated ability to delineate tumor borders and monitor temperature rise, thus enriching spatial precision and avoiding unwanted off-target effects.

Although MRI guidance provides the best resolution for thoroughly identifying and targeting lesions, it unfortunately comes with significant infrastructural needs as well as clinical and cost burdens. US guidance has thus rapidly emerged as a less costly, less encumbered, more readily portable alternative approach—albeit, at a cost to spatial precision and real-time thermography. US-guided T-FUS has been heavily investigated across various preclinical and clinical settings for targeting primary breast tumors (33-35).

In addition to MRI and US imaging, HMI has emerged as a promising alternative imaging modality for detecting cancerous lesions and performing real-time assessment of mechanical properties in breast tissues. HMI is an imaging technique that utilizes FUS to induce vibrations at the specific tissue location being examined. HMI achieves this by using a FUS transducer that generates an amplitude-modulated signal that then creates oscillations or vibrations in the tissue at the focal point of the ultrasound beam. These induced vibrations yield valuable information about the viscoelastic properties of tissues. The oscillation energy generated by the FUS allows for the assessment of tissue stiffness even in deep regions of the body. Notably, HMI is not affected by acoustic properties or noise during signal acquisition, as a separate imaging transducer is used to estimate tissue displacement. Additionally, HMI is less prone to artifacts caused by body movement or breathing, enhancing its reliability (34-39). There have been a handful of preclinical studies documenting use of HMI-guided FUS (HMIgFUS) in murine BC models, both transgenic (40) and syngeneic (38). These studies have demonstrated the robustness of HMIgFUS in monitoring tissue stiffness during thermally ablative treatments (38, 40, 41). HMI has also found success at the clinical level in patients with breast tumors. Investigations have demonstrated that HMI can distinguish tumor-bearing regions from healthy regions, as well as enable lesion classification (34-39); indeed; in line with breast elastography studies, HMI displacement maps have demonstrated the ability to differentiate between malignant and benign breast tumors, with malignant tumors exhibiting higher contrast (39). Moreover, when applied to postsurgical human breast specimens, HMI in conjunction with T-FUS was effective for monitoring thermal ablation in both healthy and pathological breast tissues (39). The integration of HMI and therapeutic ultrasound has opened a promising avenue for transitioning from anatomical information to more functional information with real-time acoustic imaging guidance tools, enhancing the robustness of tissue characterization before and after treatment. There is an ongoing clinical study deploying HMIgFUS in the breast setting (albeit, in benign breast fibroadenomas), with the goal of demonstrating potential benefits in cost reduction and enabling monitoring of mechanical changes in the tumor microenvironment (TME). While the outcomes of this trial are pending, it highlights the rapidly advancing role of HMIgFUS in clinical settings and promise therein for treating malignant BC. The continued progress of ablative approaches may give rise to alternative image-guidance solutions compatible with FUS technology and well-poised for clinical application.

Numerous preclinical studies have performed histological characterization following T-FUS, noting evidence of swelling, hyperemia, and necrosis (34, 42), as well as a reduction in tumor blood flow (25). Assessment of the safety and efficacy of T-FUS has also been conducted in feline and canine subjects with mammary cancers, demonstrating no off-target effects and well-defined regions of coagulative necrosis, consistent with a general absence of adverse events (43). While adverse events can be minimal, one clinical study more deeply probed mammary tissue properties following ablation and concluded that the degree of attenuation in breast samples did not vary on the basis of the density of the sample; however, the average acoustic attenuation coefficient in samples treated with T-FUS was significantly greater than in those untreated. With this in mind, parameters such as the attenuation coefficient may provide useful information toward the complex optimization problem of determining the “best” sonication parameters (i.e., power, sonication duration, number and pattern of sonications) for various applications and even designing more optimal T-FUS devices for treatment of BCs (33).

In addition to its use in BC, thermal ablation has been employed in numerous completed and ongoing clinical trials for treating BC bone metastases (Table 1). These trials highlight the effectiveness of thermal ablation in pain palliation and improving patient quality of life (43-52). Notably, one trial compared magnetic resonance–guided FUS (MRgFUS) thermal ablation with radiotherapy in patients with various bone metastases, including those from BC. The results showed that MRgFUS reduced pain scores from 6.6 to 2.5 one week posttreatment, whereas radiotherapy reduced pain scores only from 6.2 to 4.8 (49). MRgFUS demonstrated a significantly higher response rate compared with radiotherapy, with 71% versus 26% of patients experiencing significant pain relief (49). With interest in therapeutic ultrasound steadily growing, phase 1 and 2 clinical trials are increasingly underway for investigating the safety, feasibility, and efficacy of thermally ablating with various FUS devices (Supplemental Figure 2; Table 1).

### Mechanical Ablation

In contrast with thermal ablation, mechanical ablation via histotripsy utilizes a distinct set of acoustic exposure conditions to destroy targeted tissues via fragmentation of cells into acellular debris. The use of short ultrasound bursts (i.e., microsecond scale), a low duty cycle (≤1%), and higher peak pressure amplitudes induces acoustic cavitation of endogenous tissue gasses while minimizing heat production (53) (Figure 2). Indeed, recent work investigating the cellular effects of histotripsy using three-dimensional BC tissue mimicking phantoms showed that cells experienced displacement/deformation due to bubble expansion and collapse, while also providing evidence of clear boundaries of cell fractionation and lesion formation, with cells closest to the bubbles experiencing greater damage (54). Details pertaining to the various forms of histotripsy, underlying acoustics, bioeffects, and applications have been comprehensively highlighted in a recent *Annual Review of Biomedical Engineering* article (55). Complementing preclinical findings in BC, a recent phase 1 clinical study has also demonstrated the safety and feasibility of cavitational histotripsy treatment for patients with BC liver metastases (56).

Boiling histotripsy (BH) differs from other forms of histotripsy in that it utilizes millisecond ultrasound pulses to create vapor bubbles that rapidly expand and collapse, resulting in precise mechanical disruption and emulsification of targeted tissue structures (57-59). BH has been demonstrated to be effective in mechanically fragmenting BCs and inducing tumoricidal responses in vitro (59) and in vivo (57, 58). Studies have demonstrated that, following BH treatment in syngeneic (57) and orthotopic (58) murine 4T1 BCs, distinct regions of complete liquefaction and ablation were evident in the focal zone, with the periablative zone containing acellular fragments. Much of the research pertaining to histotripsy has been directed toward other solid tumor types. While it holds promise due to its ability to precisely ablate tissue without significant thermal damage, specific clinical trials evaluating the various forms of histotripsy in BC will be needed to explore safety, feasibility, and therapeutic utility. Notably, recent work has demonstrated the feasibility of BH in ex vivo clinical BC specimens (60).

### Pulsed Low-Intensity Focused Ultrasound

The mechanical effects of FUS can also be amplified via the concomitant administration of MBs. The pairing of low-intensity pulsed FUS and MBs has been applied to transiently open the blood–brain barrier (BBB) and blood–tumor barrier (BTB) in brain tumors, including BC brain metastasis (BCBM). As previously described, systemically circulating MBs amplify mechanical effects in an acoustic field through cavitation activity. The biological effects of cavitation are highly dependent on whether MBs are oscillating in a linear (i.e., stable) or nonlinear (i.e., inertial) regime. While stable cavitation is canonically considered to confer safe and reversible mechanical effects, inertial cavitation can lead to irreversible membrane damage, altered cell metabolism, and, occasionally, cell death (16, 61). The application of MB-assisted FUS and the role of cavitation have been studied extensively for enhancement of therapeutic delivery to BCBMs and other brain pathologies (62-66) (Figure 2). Aside from BBB/BTB opening (BBB/BTB-O) applications, a distinct set of exposure conditions referred to as pulsed FUS (pFUS) with MBs has been evaluated for peripheral applications, such as NP delivery; for example, in MCF-7 BC spheroids, it was found that anionic carboxylate particles penetrated the core of spheroids to a greater extent compared with neutral or cationic particles. This trend remained consistent as exposure to pFUS increased (30, 60, and 90 s) (67). In separate applications, pFUS has been used without the assistance of MBs, mediating sublethal mechanical forces capable of altering cellular membrane permeability and inducing inflammatory responses in the targeted region (68-72). In later sections of this article, titled Mechanical Immunostimulation and Focused Ultrasound Applications in Brain Metastasis of Breast Cancer, applications of BBB/BTB-O and pFUS across preclinical and clinical settings are discussed in greater depth.

### Sonodynamic Therapy

A limitation of the earlier highlighted FUS regimens is that they largely fail to offer cellular specificity; rather, they target precise tissue volumes in bulk. Sonodynamic therapy (SDT) offers a unique, emerging strategy for inducing tumor cell–specific cytotoxicity. SDT involves the use of sonosensitizing agents that preferentially accumulate in tumor tissue that can then be activated by exposure to low-intensity sound waves, resulting in a chemical reaction that liberates reactive oxygen species (ROS) (73), among other mechanisms, to collectively yield cellular damage and apoptosis in a localized and specific manner (Figure 2).

Sonosensitizers can be categorized as either organic or inorganic compounds that become reactive under the influence of ultrasound. Common organic sonosensitizing agents used in BC include chlorin e6 (Ce6) (74), Rose Bengal (RB) (75), and 5-aminolevulinic acid (5-ALA) (76), whereas inorganic sonosensitizers include materials such as mesoporous silica NPs, titanium dioxide (TiO_2_), and manganese dioxide (73). There have been numerous in vitro (73, 74, 76-80) and in vivo (73, 74, 76-78, 80) studies evaluating the therapeutic benefit of SDT using various sonosensitizers, with or without additional incorporation of photodynamic therapy (PDT). A collection of studies determined that the antitumoral effects of SDT using 5-ALA or hypocrellin B (79) are mediated via mitochondrial oxidative damage (76). Meanwhile, sonosensitization with IR-780 (78) or RB4 (80) has also been shown to inhibit tumor growth in 4T1 and MDA-MD-468 tumors, respectively.

Numerous studies have evaluated the antitumor efficacy of inorganic sonosensitizers (81-87) in combination with SDT. One notable study developed a novel cancer cell membrane (CCM) biomimetic nanoplatform based on hollow mesoporous TiO_2_ NPs (HMTNPs) loaded with hydroxychloroquine sulphate (HCQ) (an autophagy inhibitor), also referred to as CCM-HMTNPs/HCQ. In vivo results in MCF-7 BC-bearing nude mice demonstrate the combinatorial efficacy of CCM-HMTNPs/HCQ and SDT, which yielded the largest reduction in tumor outgrowth relative to controls. Following treatment, H&E and TUNEL staining showed significant cellular apoptosis in the CCM-HMTNPs/HCQ + SDT group compared with other groups (86). Another study introduced a novel sonosensitizer, MnWO_*X*_, encapsulated within poly(ethylene glycol) (PEG) NPs (MnWO_*X*_-PEG NPs). In vitro findings demonstrated enhanced production of O_2_ and OH under SDT conditions. Moreover, in vivo (4T1) experiments showed substantial inhibition of tumor growth relative to control groups, irrespective of whether NPs were administered intravenously or intratumorally (85). Comparatively, another study investigated the efficacy of leveraging tumor exosomes (EXO) housing sinoporphyrin sodium (DVDMS). The goal was to use EXO as a strategy for improving drug stability and enhancing drug accumulation within primary and metastatic tumors. In vitro results showed specificity toward BC cells, with the greatest efficacy in 4T1 cells compared with other cell lines including NIH/3T3, MCF-7, MDA-MB-231, and CT26. ROS production was measured using red dihydroethidium (DHE) fluorescence, which showed a significant fluorescent signal upon stimulation with SDT. Further investigation in 4T1 tumor–bearing mice showed significant reduction of tumor progression when EXO-DVDMS was stimulated with SDT compared with other groups (83). These results highlight the potential antitumoral effects of NP-formulated sonosensitizers under SDT stimulation. However, there have also been reports on the efficacy of combining SDT and PDT. Notably, studies using sonosensitizers such as Ce6 (74) and DVDMS (77) in murine BC showed significant suppression of tumor growth and lung metastasis upon SDT and PDT costimulation.

While preclinical data suggest encouraging therapeutic benefits of SDT for BC, more clinical research is needed to fully understand its potential. A case report of a BC patient who underwent SDT in combination with hormonal treatment and Gc protein–derived macrophage-activating factor (GcMAF) showed promise, with no serious adverse effects reported except for minor joint pain from the hormonal therapy (88). However, significant translational efforts are needed in this space.

### Radiosensitization

In recent years, FUS has also emerged as a potent strategy for sensitizing solid tumors to ionizing radiation, thereby enhancing the efficacy of conventional radiotherapy and enabling potential for dose de-escalation. MB-assisted FUS aids in sensitizing cancer cells to radiation via increased vascular permeability, decreased vascular integrity, and enhancement of the cytotoxic effects of radiation (89, 90) (Figure 2). This is highlighted in the example of a preclinical investigation utilizing MDA-MB-231 xenografts, wherein a mechanical FUS regime with MBs improved efficacy of radiotherapy treatment (89). Outside of preclinical findings, clinical investigations treating patients with FUS + MBs alongside radiotherapy are underway and have not unveiled any significant toxicities or off-target effects to date (90-92). One phase 1 clinical study treated 8 patients with 9 tumors, demonstrating that among the 7 patients who completed the study, all tumors showed a complete response at the treated site. Despite achieving a complete response at the treated site, these patients ultimately succumbed to metastatic burden due to the advanced stage of metastatic disease at the initiation of treatment (90). Results from another recent phase 1 clinical study recapitulated these findings, demonstrating the efficacy of combining FUS + MBs with radiotherapy, with 83% of tumors achieving partial or complete response among the 20 patients treated (Table 1) (92). Taken together, these studies underscore a promising role for radiosensitization as a strategy for both improving radiotherapy efficacy and reducing its toxicity burden through reduction of the required radiation dose.

## MODULATION OF IMMUNE RESPONSES WITH FOCUSED ULTRASOUND

### Thermal Immunostimulation

Aside from their direct physical impact, thermal FUS regimens have also been shown to elaborate immune responses (93, 94). FUS-induced hyperthermia can induce the release of HSPs, such as HSP70 (93), and, outside of BC, FUS hyperthermia has elicited immunomodulatory effects in other tumor models (95, 96). For instance, one of the earliest incidental discoveries of this phenomenon was in a 1994 study treating human posterior choroidal melanoma tumors with FUS hyperthermia. One week following treatment, there was a recalibration of the CD4^+^/CD8^+^ T cells ratio in all patients, suggesting underlying alterations to the immune response from hyperthermia treatment alone (97).

Similar to hyperthermia, T-FUS has demonstrated broad immunomodulatory effects and capacity for eliciting antitumor immune responses, as shown in both preclinical (26) and clinical (98-101) studies. Preclinical research indicates that T-FUS enhances the release of proinflammatory cytokines, such as interleukin 6 (IL-6) and IL-1β (26). Other work has shown that T-FUS increases the population of CD8^+^ T cells and reduces regulatory T (T_reg_) cells within the tumor (42), while also enriching the number of dendritic cells (DCs) in the tumor-draining lymph nodes (TDLNs). Furthermore, T-FUS has been shown to increase both the number and percentage of activated (CD11c^+^/CD86^+^) DCs in the TDLNs (34).

In the context of clinical findings, a phase 1 study revealed significant upregulation of HSP70 and epithelial membrane antigen (EMA) following T-FUS in treated tumors. These results highlight the potential of T-FUS to enhance differentiation markers and elaborate tumor immunogenicity (99). Additional clinical work has demonstrated that T-FUS can induce significant infiltration of antigen-presenting cells (APCs) (i.e., DCs, macrophages, and B cells) in the periablative margins of breast tumors (101), as well as confer increases in local and systemic CD4^+^ and CD8^+^ T cells (98, 100). Observations of enrichment in B cells and natural killer (NK) cells in the TDLNs have also been made following thermal ablation (98). Intratumoral CD4^+^ and CD8^+^ T cells have been shown to display significant increases in FasL, granzyme, and perforin expression, consistent with T cell activation and cytotoxicity (94). APCs in T-FUS settings have displayed significant increases in expression of maturity/activation markers such as HLA-DR, CD80, and CD86 (101). These results underscore the immunomodulatory potential of thermal FUS treatments. There is now growing interest in this concept, as evidenced by multiple clinical trials (NCT03237572, NCT04796220, and NCT04116320) that are designed to explore the impact of thermal FUS on the local and systemic immune landscape in BCs (98, 100).

### Mechanical Immunostimulation

Mechanical FUS regimes have also been widely studied for their immunomodulatory effects in preclinical models of BC. Mechanical FUS has been shown to increase the release of interferon gamma (IFN-γ) as well as induce greater intratumoral infiltration of CD4^+^ T cells, CD8^+^ T cells, and NK cells. Furthermore, mechanical FUS has been shown to augment the proliferation and activation states of CD4^+^ and CD8^+^ T cells posttreatment, specifically leading to the increase of Ki67 expression as well as increased CD69^+^ expression and granzyme production in the CD8^+^ T cell compartment. The reported innate immune effects of mechanical FUS have included increases in DC maturation through the upregulation of major histocompatibility complex II (MHC-II) and CD80, as well as suggestions of macrophage repolarization (104).

BH has been shown to increase M1 macrophage-associated markers (e.g., IFN-γ, CD68, IL-6, MMP9, and IL-1β) and decrease M2 macrophage markers (e.g., CD163, IL-10, and CD200R1), as determined by gene expression levels following in vitro treatment of 3D BC spheroids (59). Decreases in M2 macrophages (CD11b^+^F4/80^+^CD206^+^) (57) and CD8^+^ T cell increases (57) have also been observed following BH treatment (48). One study also directly reported the enrichment of damage-associated molecular patterns (e.g., calreticulin, HSP70, high mobility group box-1) following exposure to BH; notably, similar observations have been reported for T-FUS. Other studies have implicated a role for tumor necrosis factor (TNF) signaling following BH, along with postablative increases in secretion of chemokines/cytokines such as IFN-γ, IL-1α, IL-1β, IL-18, and IL-8 (57).

pFUS has also been demonstrated to be capable of eliciting sterile inflammatory responses (68, 70, 72). Reports have showcased clear tumor inhibition following pFUS in 4T1 tumors (68, 72), where exposures were reported to increase CD4^+^ T cells, CD8^+^ T cells, DCs (68, 72), and NK cells (72) within tumors (63). pFUS has also been shown to transiently increase cytokine release (i.e., IL-1α, IL-1ß, IL-2, IL-6, IL-12p40, IL-15, IL-17) and TNF-α production, as well as to elevate vascular cell-adhesion molecule (VCAM) expression (72). Another study recapitulating these findings has offered the additional insight that increasing pFUS peak negative pressure (PNP) can in turn yield increases in cytokines, chemokines, and trophic factors [i.e., IL-10, transforming growth factor beta (TGF-ß), IL-1, IL-17] and cell-adhesion molecules. It was found that greater PNP (≥4 MPa) elicited antitumor immune signatures, with DNA damage and reductions in T_reg_ cells, tumor-associated macrophages, and myeloid-derived suppressor cells (MDSCs) observable at 6 MPa (70). Various modalities for mechanical FUS offer mechanisms to induce local and potential systemic immune effects posttreatment, providing a rationale for designing new therapeutic paradigms leveraging these effects toward more effective combinatorial BC immunotherapy.

### Immunostimulation with Sonodynamic Therapy

With the maturation of SDT paradigms across various solid tumor settings including BC, recent studies have placed a lens on the immunogenic potential of FUS in combination with various sonosensitizers. One notable investigation used phase-transformation perfluorocarbon (LIP-PFH) nanoparticles for SDT in 4T1 tumor–bearing mice, eliciting increases in T helper (T_h_) and cytotoxic T (T_cyt_) cells in peripheral blood and decreased abundance of M2 macrophages and T_reg_ cells. By immunohistochemistry, elevations in CD4^+^ and CD8^+^ T cells and a decline in programmed cell death protein 1 (PD-1) expression on tumor-infiltrating T cells were observed (84). SDT has also been shown to induce pyroptosis, an inflammatory programmed cell death mode. A recent study evaluated the immune response downstream of SDT-driven pyroptosis using LY364947-loaded erythrocyte membrane-coated PCN-244 NPs (LPM) in triple-negative BC-bearing mice. Specifically, decitabine was used to upregulate gasdermin E, which is cleaved during the initiation of pyroptosis. Findings of this study included significant antitumor efficacy, with a fraction of the mice in the combinational group exhibiting complete response. Significant increases were observed in CD80^+^/CD68^+^ DCs in TDLNs and intratumoral CD8^+^ T cells. Among the mice that experienced a complete response, there was a notable increase in central memory (CD62L^+^/CD44^+^/CD4^+^) and effector memory (CD62L^−^/CD44^+^ /CD4^+^) T cells in the spleen three weeks after rechallenge (107). Although only a handful of studies have implicated the immunomodulatory capability of SDT in BC, current insights underscore the potential of this regimen to incite immunogenic cell death mechanisms and contribute to productive immune responses.

## COMBINATION OF FOCUSED ULTRASOUND WITH IMMUNOTHERAPIES

### Combinatorial Paradigms with Ablative Focused Ultrasound Modalities

In addition to demonstrating immunomodulatory capacity, a wealth of literature has now demonstrated the promise of combinatorial therapy approaches specifically leveraging FUS for potentiation of immunotherapy drugs to which BCs are poorly responsive at baseline (Supplemental Table 2). In one preclinical BC study, the combination of a microcavitating mechanical FUS regimen with anti-PD-L1 (anti–programmed death-ligand 1) was observed to significantly enhance the antitumor response, characterized by increased populations of CD4^+^ T cells, CD8^+^ T cells, and NK cells. This combination also conferred a notable survival benefit in HER2+ BC xenografts (104). In a bilateral HER2 oncogene-dependent murine model (MM3MG-HER2), mechanical FUS monotherapy elicited a more robust cellular response characterized by activation of T cells and NK cells and constrained outgrowth of untreated contralateral tumors in comparison with T-FUS treatment. Furthermore, mechanical FUS induced stronger expression of PD-L1 on various immune cells and improved immune-mediated tumor growth control when combined with PD-1/PD-L1 blockade.

While a handful of reports like this suggest that mechanical ablation may have more favorable immunologic benefits over its thermal counterpart (57, 104), T-FUS has a long and rich history of being utilized to enhance the effectiveness of immunotherapies in BC. One study explored the combined effect of priming with Toll-like receptor agonist CpG and anti-PD-1 treatment one week prior to T-FUS application, which resulted in reduced macrophages and MDSCs, augmented IFN-γ-producing CD8^+^ T cells, and increased the proportion of M1 macrophages in BC-bearing mice (26, 42). Interestingly, this study also demonstrated that immunotherapy priming was more efficacious than concomitant initiation with FUS, offering critical insights regarding sequencing of FUS and pharmacologics in combinatory immunotherapy paradigms.

Although T-FUS has known immunomodulatory effects, these effects alone have largely proven insufficient to sustain a robust antitumoral response. Therefore, a recent study has introduced a novel combinatory approach integrating T-FUS with oxygen-carrying biomimetic perfluorocarbon NPs to induce immunogenic cell death and convert the immunosuppressive BC microenvironment into an immunologically favorable one. This combination effectively mitigated hypoxia within the TME and repolarized M2-like (protumor) macrophages into M1-like (antitumor) macrophages in 4T1-bearing mice. Incorporation of anti-PD-1 therapy into this paradigm further enhanced its efficacy, as displayed by effective inhibition of primary and distal tumors in a bilateral 4T1 model. Immunohistochemical staining revealed an enrichment of CD8^+^ T cells in the ablated primary tumor as well as the nonablated contralateral tumor, accompanied by an increase in mature DCs in primary tumor and TDLNs (108). Another study developed PEGylated poly(lactic-coglycolic) acid (PLGA) NPs encapsulating astragalus polysaccharides (APS) and gold nanorods (AuNRs), referred to as APS/AuNR/PLGA-PEG NPs. Using a co-culture of bone marrow–derived DCs from naive Balb/c mice and 4T1 cells, it was found that the proportion of MHC-II, CD86, and CD80 was significantly higher when APS/AuNR/PLGA-PEG NPs were administered prior to T-FUS. These findings suggested that APS/AuNR/PLGA-PEG NPs can induce the phenotypic maturation of DCs in vitro. Observations from in vivo studies in 4T1 tumors demonstrated that T-FUS combined with APS/AuNR/PLGA-PEG NPs could significantly upregulate TNF-α, IFN-γ, IL-4, IL-10, and immunoglobulin G1 (IgG1) (109) relative to blank control. Although the combination of T-FUS with NPs may induce meaningful immunological changes, this approach has not yet made it into clinical practice, and further studies are needed to evaluate the therapeutic efficacy of this and other NP-based approaches for elaborating robust immunity against BCs.

### Combinatorial Paradigms with Nonablative Focused Ultrasound Modalities

Other modes of FUS outside of the ablative regimes have also been examined in alliance with immunotherapies (Supplemental Table 2). For example, a study using trastuzumab-loaded liposomes (TRA-LPs) in HER2+ BC cells (SKBR3), in conjunction with mechanical FUS, demonstrated significant improvement in intratumoral drug accumulation post-FUS and resultant reduction in cell viability relative to controls (110). Another study demonstrated the intratumoral delivery of nanocomplex-conjugated MBs with ultrasound to activate cyclic GMP-AMP synthase-stimulator of interferon genes (cGAS-STING) and downstream pathways for antigen-specific T cell priming. A sonoporative ultrasound regimen was used to deliver nanocomplex-conjugated MBs bearing 2′3′-cyclic guanosine monophosphate-adenosine monophosphate (cGAMP), which was effective in controlling primary 4T1 and E0771 tumor outgrowth. Furthermore, the incorporation of PD-1 blockade into this combinatorial strategy elaborated superlative control of 4T1 secondary lung metastases, in addition to primary tumors (111).

Additional work has investigated SDT and liposomes housing the sonosensitizer HHME and imiquimod [R837, targeting Toll-like receptor 7 (TLR7)], combined with PD-L1 blockade immunotherapy. The liposomal delivery of TLR7 in combination with SDT and checkpoint blockade elaborated a strong antitumor response, evidenced by significant constraint of primary tumor growth and prevention of pulmonary metastasis (112). There have also been efforts to use thermosensitive liposomes (TSLs) with hyperthermia to improve localized immunotherapy delivery, wherein cargo release occurs when tissues are warmed to ~42°C. One study developed TSLs loaded with R848—a TLR7/8 agonist with potent antitumor and immunostimulatory activity that is poorly tolerated when delivered systemically. R848-TSL administered locally or systemically in combination with ultrasound-mediated hyperthermia resulted in tumor regression and enhanced survival when combined with αPD-1 in mice bearing *neu* deletion line (NDL) breast tumors (Her2+, ER/PR negative) (113). Another study investigated the immunomodulatory effects of FUS-induced hyperthermia in combination with DOX-loaded TSLs, joint with administration of CpG and αPD-1, in both the NDL HER2+ BC and the MMTV-PyMT transgenic models. A treatment paradigm involving priming with immunotherapy before hyperthermia and TSL-loaded DOX treatment elaborated significant intratumoral CD8^+^ T cell infiltration in both treated and distant tumors. Furthermore, 90% of the NDL mice treated with this combinational approach experienced a complete response to this therapeutic paradigm (94).

Existing reports suggest that FUS holds significant promise as a transformative adjunct to immunotherapies, offering a synergistic approach that harnesses the precision of acoustic energy to enhance immune-mediated cancer treatment. As research continues to reveal the complex interactions between FUS and immune pathways, its integration into clinical practice could significantly broaden therapeutic possibilities, ultimately improving outcomes for patients with a variety of malignancies—notably immunologically “cold” BCs.

## COMBINATION OF FOCUSED ULTRASOUND WITH CHEMOTHERAPIES

See the Supplemental Material for coverage of FUS applications in combination with free and nanoparticle-encapsulated chemotherapies.

## FOCUSED ULTRASOUND APPLICATIONS IN BRAIN METASTASIS OF BREAST CANCER

Metastasis of primary tumors to secondary sites such as the brain remains a key factor in the persistent morbidity and mortality rates associated with BCs. The task of treating brain metastases is formidable, in part owing to the challenging anatomy of the central nervous system (CNS) and the distinctly protective nature of the BBB. The BBB is a dense vascular network composed of endothelial cells, pericytes, and astrocytes that surround and protect the brain. The BBB is impermeable to nearly 100% of large-molecule (>500 Da) drugs and 98% of small-molecule drugs. The presence of primary or metastatic brain tumors gives rise to the additional presence of the BTB, which is characterized by leaky vasculature, due to increased vascular endothelial growth factor (VEGF) expression, and loss of BBB tight junctions along the endothelium. While the BTB and destabilized BBB within brain tumors can make them more permeable, this heightened permeability is often insufficient for drugs to effectively deposit within brain tumors—owing to other counterproductive characteristics of the TME that limit therapeutic transport, such as stromal barriers and high interstitial fluid pressures (114). In this section, we highlight BCBMs as an exemplar of how transcranial FUS technology is being used to overcome limitations to CNS cancer-targeted therapy.

### Antibody Delivery

Trastuzumab is among the standard-of-care interventions for HER2+ BCBMs, despite limited penetrance across the BBB/BTB, much like many macromolecular biologics of similar size and structure (115). Transient, reversible FUS BBB/BTB-O across the intact skull has emerged as an attractive noninvasive option for augmenting therapeutic delivery to the CNS. A handful of studies have used FUS BBB/BTB-O to enhance the delivery of trastuzumab and/or pertuzumab to BCBMs (65, 66, 116, 117). In one example, deploying a 690-kHz MRgFUS system for BBB/BTB-O at either 0.6 or 0.8 MPa in murine BCBM xenografts enhanced delivery of trastuzumab; notably, the concentration of antibody was significantly higher at the higher PNP, more than twice that at the lower PNP (65). Investigative efforts in a HER2+ human breast carcinoma (BT-474) xenograft in rats have shed light on responder/nonresponder dichotomies in this therapeutic context, finding that trastuzumab delivery with FUS BBB/BTB-O reduced tumor outgrowth in some (4 out of 10) rats but conferred only a mild response in others (6 out of 10) (64). While the growing body of preclinical work in this space underscores the importance of parameter tuning for optimization of antibody access, it has more importantly laid the foundation for ongoing clinical trials investigating FUS in combination with trastuzumab.

**Figure 4:**
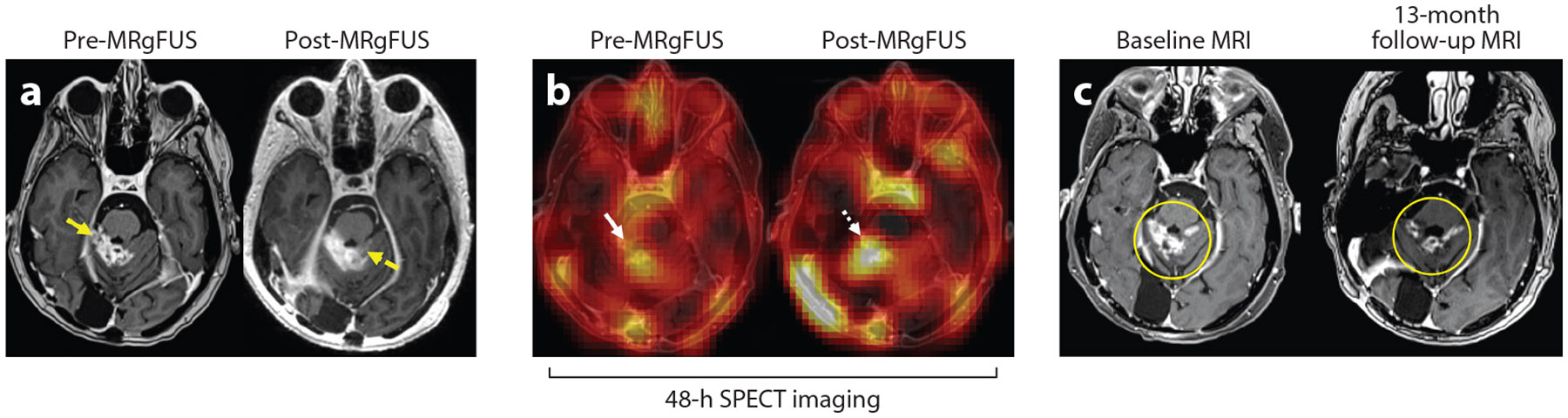
Visual demonstration of antibody delivery across the BBB/BTB of BCBM with MRgFUS. (*a*) Depictions of BCBM on T1-weighted MRI pre- and post-MRgFUS BBB/BTB-O. Yellow arrows denote contrast enhancement in the tumor and surrounding region before and after sonication. (*b*) SPECT imaging 48 h pre- and post-MRgFUS BBB/BTB-O following delivery of ^111^In-BzDTPA-NLS-trastuzumab. White arrows denote increase in the SPECT signal in the region targeted with FUS, suggesting increased antibody delivery. (*c*) BCBM (*yellow circle*) on baseline scan prior to therapeutic intervention and on follow-up scan taken 13 months after treatment. Follow-up imaging exhibits reduction in tumor burden 13 months after FUS + trastuzumab treatment. Abbreviations: BBB, blood–brain barrier; BBB/BTB-O, BBB/BTB opening; BCBM, breast cancer brain metastasis; BTB, blood–tumor barrier; FUS, focused ultrasound; MRgFUS, magnetic resonance–guided FUS; MRI, magnetic resonance imaging; SPECT, single-photon emission computed tomography. Figure adapted with permission from Reference 62.

A recent first-in-human study provided the first visual demonstration of antibody delivery across the BBB/BTB with MRgFUS, notably with trastuzumab delivery to patients with HER2-enriched brain metastases (Figure 4) (62). This has since scaled into a larger ongoing clinical trial seeking to treat brain metastases with trastuzumab aided by MRgFUS. Yet another study has explored the combinatory effect of trastuzumab and pertuzumab following BBB/BTB-O in immunocompromised rats bearing HER2+ MDA-MB-361 xenografts. MRgFUS BBB/BTB-O was repeated weekly alongside administration of these antibodies, and partial response to FUS combined with trastuzumab/pertuzumab was noted (66).

Trastuzumab has also been examined as an immunotherapeutic for treatment of leptomeningeal metastases (LMs) associated with BC. In addition to the aforementioned CNS barriers, LMs are constrained by an additional physical barrier known as the blood–spinal cord barrier (BSCB), which canonically restricts molecules greater than 500 Da in size. In an athymic nude rat model bearing spinal cord LMs derived from a HER2+ BC line (MDA-MB-231-H2N), FUS was demonstrated to mediate delivery through the BSCB. In this pilot study, trastuzumab and MB-assisted FUS were concomitantly applied on a weekly basis for three weeks, with antibody dosage starting at 8 mg/kg in the initial week and undergoing de-escalation to 6 mg/kg for subsequent dosages. Although technical factors limited interpretations, tumor growth suppression was qualitatively observed on MRI in the FUS-recipient group and further confirmed by tumor volume measurements on histology, lending promise to the use of FUS as a strategy for overcoming the BSCB in leptomeningeal disease (117).

### Antibody–Drug Conjugates and Adoptive Cellular Therapy Delivery

The treatment of HER2+ BCBMs with trastuzumab has also been explored in the context of antibody-drug conjugates (ADCs). ADCs are an emerging therapeutic class consisting of a tumor-specific monoclonal antibody attached to a cytotoxic drug through chemical linkage. The cytotoxic drug remains in an inactive form until internalized by a tumor cell. Recent work has investigated FUS for improving the delivery of an ADC, ado-trastuzumab emtansine (T-DM1), across the BBB—as well as DOX—in an orthotopic xenograft model of HER2+ BCBM. These compounds were independently assessed for their extravasation and penetrance into BCBM lesions. Following FUS BBB/BTB-O, DOX and T-DM1 exhibited sevenfold and twofold increases in extravasation, respectively—an observation consistent with the size of each molecule. Additionally, immunostaining revealed increased penetrance into the tumor bulk for each of these agents following FUS BBB/BTB-O (63).

In addition to therapeutic antibodies and ADCs, FUS has also been evaluated in combination with adoptive cellular therapies. In the context of BCBMs, preclinical work has demonstrated the successful delivery of human NK cells across the BBB/BTB with FUS in orthotopic HER2-amplified BCBMs. Injection of NK-92 cells before FUS resulted in an approximately fivefold increase in the ratio of effector to tumor cells, demonstrating enhanced delivery of adoptively transferred cells to the brain with FUS BBB/BTB-O (118). In another study, an early intensive treatment paradigm combining HER2-targeted NK-92 cells with FUS in a front-loaded manner yielded improved survival of BCBM-bearing rats in comparison with a distributed treatment paradigm; early intensive treatments conferred long-term survival in 50% of subjects (119).

### Nanotherapeutic Delivery

Much as nanotherapeutics have been an area of rapid development for primary BC treatment with FUS, emerging work is fortifying the promise of nanotherapeutic-aided FUS paradigms in BCBMs. Reports demonstrate the enhanced delivery of DOX-encapsuled PEGylated liposomes (PLDs) into the brain tumor following pulsed-wave low-dose hyperthermia and short-time FUS hyperthermia treatments (120, 121). Unlike FUS hyperthermia, pulsed-wave hyperthermia applies short bursts of ultrasound energy to the tumor site to systematically raise the local temperature to 42°C, with the goal of enhancing tissue and vascular permeability to confer nanodrug accumulation within the tumor. With these exposure conditions, tumor burden was significantly constrained in 4T1-luc–bearing mice after treatment with PLDs and FUS (120, 121). Polymeric NP approaches have also been deployed in BCBM, as in the example of a study delivering PEG-coated gold NPs (PEG-AuNPs) across the BBB/BTB with FUS in rats. The results showed a significant increase in gold content in the sonicated right hemisphere of the brain as compared with the nonsonicated contralateral hemisphere; MRgFUS exposure yielded a threefold enhancement in AuNP delivery (122).

## OUTLOOK AND PERSPECTIVES

BC persists as the most frequently diagnosed cancer in women, with the occurrence of metastases drastically amplifying its morbidity and mortality rates. Even in the remarkable instances of complete remission, significant survivorship burdens remain for patients in the aftermath of aggressive therapeutic regimens that harbor risk and toxicities. FUS offers a powerful multimodal strategy for debulking, modulating, and potentiating therapeutic delivery to both primary and metastatic BC lesions in a noninvasive, nontoxic manner.

Given the unparalleled promise of immunotherapies in cancer, emerging evidence for the immunomodulatory potential of various ablative and nonablative FUS regimens in BC is encouraging, suggesting the intriguing prospect for aligning FUS with various immunotherapeutic categories even beyond conventional checkpoint blockade. While some reports have offered suggestions of abscopal effects following FUS, further work is needed on the refinement of acoustic exposure conditions, therapeutic sequencing, and other critical parameters to fully realize this exciting potential on a more controllable and reproducible basis.

The combination of FUS with the mainstays of BC care, including surgery, radiotherapy, and chemotherapy, has proven to be an additionally promising and rapidly ripening avenue for promoting treatment efficacy and/or curbing invasiveness and toxicity. Given the burgeoning role of combinatorial therapies in cancer treatment, there is an indisputable need for strategies that can enable system dose de-escalation of therapies or elicit synergies that enable less aggressive treatment. Where preclinical data are ripe, early-phase and window-of-opportunity trials will offer a quantum leap in discovery to this end. Recent preclinical work demonstrated that the combination of thermally ablative FUS with gemcitabine could substantially improve control of aggressive BCs and in an adaptive immunity–dependent manner; this combination also sensitized these treatment-refractory tumors to PD-1 blockade, underscoring the potential for FUS to have a role in increasingly complex combinatory interventions (34). These insights laid the groundwork for an ongoing clinical trial that is now interrogating the immunological impact of low-dose gemcitabine in combination with T-FUS in a treat-and-resect design (NCT03237572). It is expected that examples of bench-to-bedside translation of this manner will continue to grow in coming years.

Notably, findings on immunotherapy delivery with FUS bear clear extension to other emerging immunotherapy classes, including chimeric antigen receptor (CAR) T cell therapy. To date, no published studies have evaluated FUS for CAR T cell therapy in BCs, but it stands to reason that FUS could play a significant role in overcoming barriers to their delivery and persistence through lifting of physical barriers and modulation of vascular activation state, secreted factors, and cellular immune landscape. As showcased by an above-highlighted study that leveraged superparamagnetic iron oxide NP loading to enable MRI surveillance of adoptively transferred NK cells, theranostic approaches could be a powerful—if not imperative—asset to deconvolving the impact of FUS on targeted delivery of complex biologics as well as their systemic or local activity (118).

Specific applications aside, FUS has been rapidly advancing as a general interventional tool in primary BCs and BCBMs with the growing availability and adoption of a diverse milieu of devices offering distinct image-guidance and real-time feedback capabilities. This has given way to a rich and evolving tapestry of completed or ongoing clinical trials worldwide that are anticipated to yield invaluable novel insights over the coming years, with relevance not only to BC but also to various other solid tumor types.

To conclude, FUS has seen a remarkable evolution as an intervention in the breast tumor setting. This began with straightforward efforts to achieve complete ablations for nonsurgical debulking of tumors and rapidly blossomed into the highly complex modern endeavors to manipulate biology. FUS is increasingly being deployed in sophisticated combinatory approaches spanning both ablative and subablative modes. Moreover, the field is seeing the advent of novel mechanisms of action (e.g., radiosensitization, SDT), novel areas of fundamental discovery (as in the case of immunomodulation), and even intersections of acoustics with other emerging disciplines such as synthetic biology [as in the case of sonogenetics, not discussed herein (123)]. To this end, FUS holds immense promise for revolutionizing not only BC management but also cancer management at large—offering a noninvasive, precise, and versatile treatment option that enhances the effectiveness of both traditional and novel treatments and stands to herald a new era of personalized cancer care.

## Supplementary Material

Supplemental Figure 1

Supplemental Figure 2

Supplemental Material and Tables

## Figures and Tables

**Table 1:** Clinical trials evaluating FUS in primary or metastatic (brain, bone, liver) breast cancers for ablation, combinatorial therapy, or pain palliation

FUS Modality	Reference(s)	Device/modality	Additional therapeutics	Site	Number of patients	Key findings	Status
**T-FUS**	Wu et al. 2003 (124)	Model-JC HAIFU USgFUS	Chemotherapy, radiation, and hormonal therapy	Breast	48	Coagulative necrosis was achieved in all patients, with only mild local pain experienced in 14 patients.	Completed
	Gianfelice et al. 2003 (29, 125, 126)	ExAblate 2000 MRgFUS	NA	Breast	12, 24, 17	Treatment was well tolerated in a majority of patients, with minor burns reported in a small fraction of patients.	Completed
	Zippel & Papa 2005 (127)	ExAblate 2000 MRgFUS	Postoperative radiation	Breast	10	Two patients had a complete response, with no residual tumor remaining post MRgFUS. Two patients had microscopic foci of residual carcinoma, 3 had 10% residual tumor, and 3 had 10–30% residual tumor. No infection or complications were reported.	Completed
	Wu et al. 2005 (35)	Model-JC HAIFU USgFUS	Chemotherapy, radiation, and tamoxifen	Breast	22	Complete coagulative necrosis was seen in all treated patients. Although no skin burns, bleeding, or infection were observed, 14 patients experienced mild local pain. The 5-year disease-free survival and recurrence-free survival rates were 95% and 89%, respectively.	Completed
	Wu et al. 2007 (99)	Model-JC HAIFU USgFUS	Chemotherapy, radiation, and tamoxifen	Breast	23	Thermal ablation of tumors was safe and successful in all patients; validation with H&E and NADH histological stains provided reliable assessment after T-FUS treatment. Cellular membrane staining of epithelial membrane antigen, CD44v6, and heat shock protein 70 were 100%, 0%, and 100%, respectively.	Completed
	Khiat et al. 2006 (31)	ExAblate MRgFUS	At least three months since prior chemotherapy and immunotherapy; no prior external radiotherapy or laser therapy to ipsilateral breast	Breast	25	Out of 25 patients, 7 tumors showed no detectable cancer cells at the targeted region, and 11 patients had residual cancer below 10%.	Completed
	Furusawa et al. 2006 (28)	ExAblate 2000 MRgFUS	No prior treatment with radiation or local thermal therapy	Breast	30	Treatment was well tolerated, with a mean necrosis rate for targeted breast tumor of 96.9%.	Completed
	Wu et al. 2007 (128)	Model-JC HAIFU USgFUS	Chemotherapy, radiation, and hormone therapy	Breast	23	Coagulation necrosis was confirmed in all 23 patients. Only 14 patients experienced mild pain after T-FUS treatment.	Completed
	Furusawa et al. 2007 (27)	ExAblate 2000 MRgFUS	NA	Breast	21	All treatments were successful, with 4 out of 21 patients receiving a second session of T-FUS. Only two cases of second-degree burns following treatment were reported.	Completed
	Kim et al. 2010 (32)	Model-JC HAIFU USgFUS	Neoadjuvant chemotherapy for tumors greater than 50 mm	Breast	6	Subtracted MRI scans showed subtle patterns of enhancement of coagulative necrosis. Furthermore, the appearance of a thin rim of enhancement may correlate with an inflammatory reaction in response to complete T-FUS treatment. Conversely, nodular or irregular thick rim enhancement may correlate with partial ablation.	Completed
	Napoli et al. 2013 (46)	ExAblate 2100 MRgFUS	Chemotherapy	Bone	18	No adverse events were recorded. Five out of 18 patients were found to have an increase in bone density with restoration of cortical border. Two patients experienced complete response and 4 patients experienced partial response posttreatment.	Completed
	Hurwitz et al. 2014 (44)	ExAblate MRgFUS	Chemotherapy, radiation, and/or hormonal therapy	Bone	112	32.1% of patients experienced treatment-related adverse events during sonication. 60.3% of adverse events were resolved on the treatment day. The mean reduction of pain scale scores was 3.6 for FUS and 0.7 for the placebo group.	Completed
	Cavallo Marincola et al. 2014 (129)	ExAblate 2100 MRgFUS	NA	Breast	5	Three out of 5 patients had confirmed 100% necrosis of the tumor. One patient had 15% residual tumor and another had 70% residual tumor postablation.	Completed
	Gu et al. 2015 (52)	ExAblate 2100 MRgFUS	No prior chemotherapy or radiation treatment	Bone	23	Three out of 23 patients experienced pain during sonication. Pain scores decreased from 6.0 to 3.7 one week posttreatment and to 2.2 three months posttreatment.	Completed
	Guan & Xu 2016 (130)	Model-JC HAIFU USgFUS	Chemotherapy, radiation, and hormonal therapy	Breast	25	Histological findings revealed distinct regions of coagulative necrosis and damage to the associated tumor vasculature in all patients. Elastin fibers were also damaged posttreatment. Staining confirmed decreased levels of VEGF expression. Electron microscopy analysis further confirmed a complete loss of integrity in the tumor capillary ultrastructure, with the capillary endothelial cells being destroyed.	Completed
	Merckel et al. 2016 (131)	Sonalleve MRgFUS	Neoadjuvant systemic chemotherapies	Breast	10	Six patients experienced maximum tumor necrosis posttreatment. No redness or burns were observed in any of the patients.	Completed
	Lee et al. 2017 (49)	ExAblate 2000 MRgFUS	No previous local therapy to bone lesion prior to MRgFUS treatment Chemotherapy, targeted therapy, bone-targeting agents, and hormonal therapy	Bone	63	FUS was compared with radiation therapy. Pain palliation was evaluated one week posttreatment. FUS reduced pain scores from 6.6 to 2.5 one week post-FUS. Pain scores were reduced from 6.2 to 4.8 one week after radiation therapy. FUS had a significantly higher response rate compared with radiation therapy (71% versus 26%).	Completed
	Bertrand et al. 2018 (45)	ExAblate 2000 MRgFUS	Exhausted maximum radiotherapy	Bone	17	37.5% of patients experienced complete response, and 50% of patients experienced partial response. Pain was found to be reduced posttreatment.	Completed
	Payne et al. 2021 (30)	Custom breast-specific MRgFUS (developed in collaboration with the Univ. of Utah and Image Guided Therapy, Inc.)	NA	Breast	10	Using novel hardware, an advanced tumor-targeting system, and a volumetric MRI protocol, treatments were achieved in 8 out of 10 patients. The remaining 2 patients experienced technical failures due to MRI sequencing errors and networking issues with the MRI scanner. No pain was reported by any of the patients.	Completed
	Hsu et al. 2022 (51)	ExAblate 2000 MRgFUS	Chemotherapy and bone-targeting agents during the period from two weeks before to three months after FUS treatment	Bone	20	Sixteen out of 20 patients experienced complete response within three months, and 4 out of 20 patients exhibited partial response. IP-10 and IL-6 were significantly reduced 24 h after thermal ablation.	Completed
	Bongiovanni et al. 2022 (48)	MR-HIFU	NA	Bone	12	Six out of 12 patients had a complete response to constant pain, while 5 out of 12 and 7 out of 12 patients had a complete response and partial response to breakthrough cancer pain, respectively.	Completed
	NCT00008437	MRgFUS	At least three months since prior chemotherapy; no prior external radiotherapy or laser therapy to ipsilateral breast	Breast	45	TBD	Completed
	NCT03342625 (BRIFU)	Custom breast-specific MRgFUS (developed in collaboration with the Univ. of Utah and Image Guided Therapy, Inc.)*	NA	Breast	15	TBD	Unknown
	NCT05350059 (HIFUB)	Undisclosed HIFU device	NA	Breast	15	TBD	Unknown
	NCT02407613	Philips Sonalleve MR-HIFU breast tumor therapy system	No prior treatment with neoadjuvant systemic therapy in past three months or radiotherapy, thermal therapy, or surgery in targeted breast	Breast	10	TBD	Early closure
	NCT05219695	HMIgFUS	No prior treatment with systemic or thermal therapy	Breast	36	TBD	Ongoing
	NCT05291507	MUSE MRgFUS system*	No prior treatment with neoadjuvant systemic therapy or radiotherapy	Breast	34	TBD	Ongoing
**FUS + Pharmacologics**	Meng et al. 2021 NCT03714243 (62)	ExAblate Model 4000 Type 2 “Neuro-System” MRgFUS	Trastuzumab	Brain	20	No treatment-related serious adverse events were reported. Effective delivery of trastuzumab with FUS was confirmed via ^111^In-BzDTPA-NLS-trastuzumab SPECT imaging. Treated lesions saw an average increase in standardized uptake value ratio of 101 ± 71%, relative to −18 ± 26% in control lesions.	Completed
	Inui et al. 2014 (88)	Undisclosed FUS device (SDT)	GcMAF + Ce6 + 5-ALA + hormone therapy	Breast	1	No serious side effects were observed following treatment. The axillary tumor, pleural effusion, and intrapleural nodular tumor in the right lung all showed a complete response to the treatment.	Completed
	NCT03477019	Undisclosed FUS device	Sonovue MBs + conventional chemotherapy	Liver	17	TBD	Completed
	NCT03749850	Philips Sonalleve MR-HIFU breast tumor therapy system	LTLD + cyclophosphamide	Breast	12	TBD	Completed
	NCT04431674 (USmBRT-B)	MRgFUS	Definity MBs + radiotherapy (LINAC)	Chest wall and breast	20	TBD	Completed
	NCT04116320 (AM-003)	Theraclion Echopulse USgFUS	Pembrolizumab + pICLC	Mixed solid tumors (including breast)	5	TBD	Early closure
	NCT03237572 (BR48)	Theraclion Echopulse USgFUS	Pembrolizumab	Breast	13	TBD	Early closure
	NCT04796220 (BR54)	Theraclion Echopulse USgFUS	Low-dose gemcitabine	Breast	48	TBD	Ongoing
	NCT05491694 (HIFU)	Undisclosed HIFU device	Toripalimab + chemotherapy (epirubicin + cyclophosphamide → nab-paclitaxel + carboplatin)	Breast	20	TBD	Ongoing

Asterisks (*) refer to the use of analogous systems under different names.

Abbreviations: 5-ALA, 5-aminolevulinic acid; Ce6, chlorin e6; FUS, focused ultrasound; GcMAF, Gc protein–derived macrophage-activating factor; H&E, hematoxylin and eosin; HIFU, high-intensity focused ultrasound; HMIgFUS, harmonic motion imaging–guided FUS; IL-6, interleukin 6; IP-10, interferon gamma-induced protein 10; LINAC, linear accelerator; LTLD, lyso-thermosensitive liposomal doxorubicin; MB, microbubble; MRgFUS, magnetic resonance–guided FUS; MRI, magnetic resonance imaging; NA, not applicable; NADH, nicotinamide adenine dinucleotide; NCT, National Clinical Trial; pICLC, polyinosinic-polycytidylic acid with poly-l-lysine and carboxymethylcellulose; SDT, sonodynamic therapy; SPECT, single-photon emission computed tomography; TBD, to be determined; T-FUS, thermally ablative FUS; USgFUS, ultrasound-guided FUS; USmBRT-B, ultrasound stimulated microbubble radiation treatment for patients with chest-wall and breast cancer; VEGF, vascular endothelial growth factor.
