# Supplementary material for: Therapeutic Ultrasound for Multimodal Cancer Treatment: A Spotlight on Breast Cancer
Source: Annu Rev Biomed Eng. Author manuscript; Available in PMC 2025 Sep 5. (PMC12411100; doi:10.1146/annurev-bioeng-103023-111151)
Supplement: Supplemental Figure 1 [file NIHMS2098925-supplement-Supplemental_Figure_1.pdf]

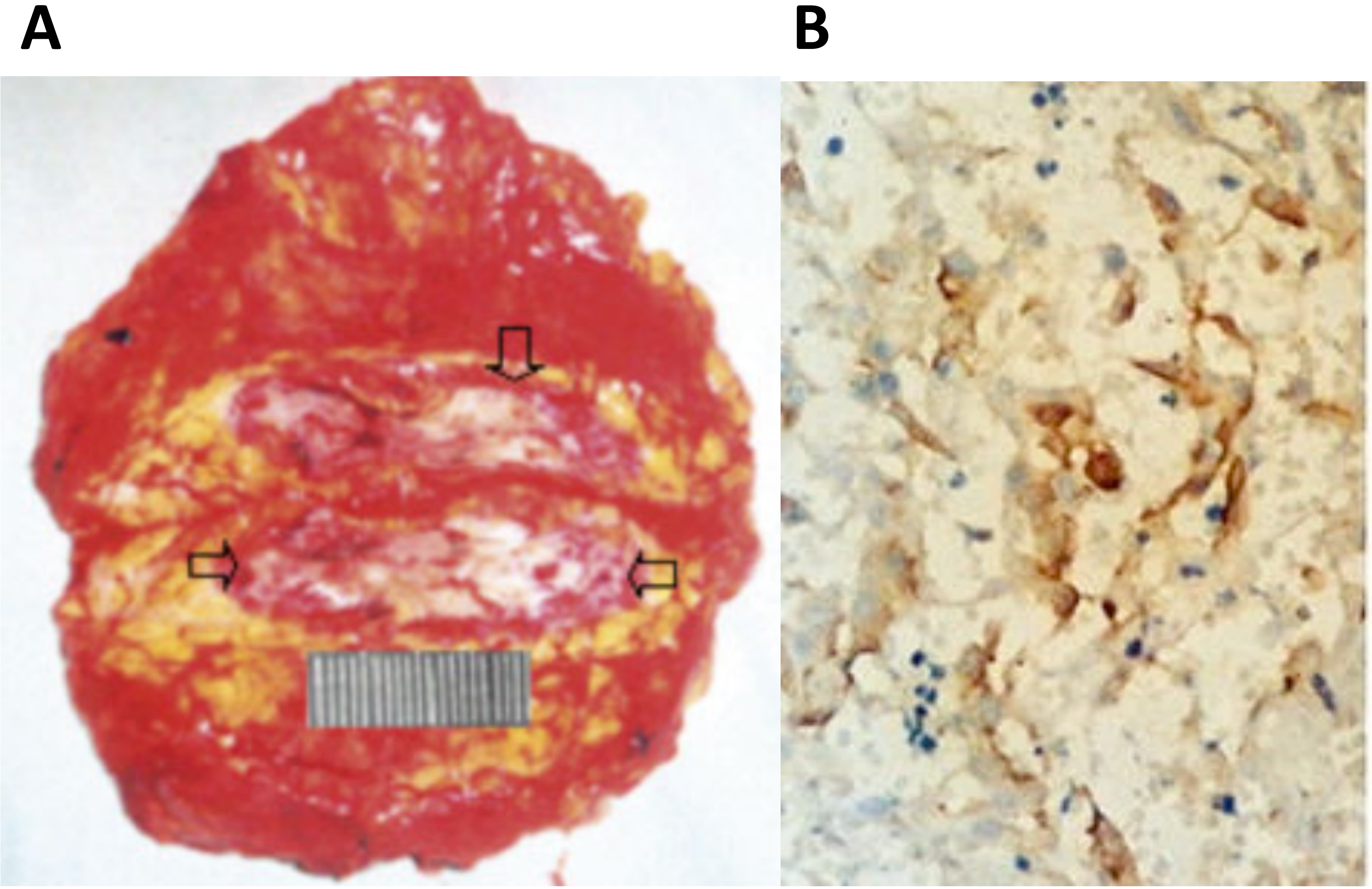

Supplemental Figure 1. Representative Breast Tumor Lesion and HSP Upregulation Following T-FUS. (A) Depiction of breast tumor following USgFUS treatment. Arrows indicate regions of coagulative necrosis. (B) Immunohistochemistry staining for heat shock protein 70 (HSP70) expression (brown stains) in tumor post-thermal ablation. Adapted with permission from (Feng Wu, et al. *J Surg Oncol.* 2007 & Feng Wu, et al. *Ann Surg Oncol.* 2007) (141, 96).
