# Supplementary material for: Therapeutic Ultrasound for Multimodal Cancer Treatment: A Spotlight on Breast Cancer
Source: Annu Rev Biomed Eng. Author manuscript; Available in PMC 2025 Sep 5. (PMC12411100; doi:10.1146/annurev-bioeng-103023-111151)
Supplement: Supplemental Figure 2 [file NIHMS2098925-supplement-Supplemental_Figure_2.pdf]

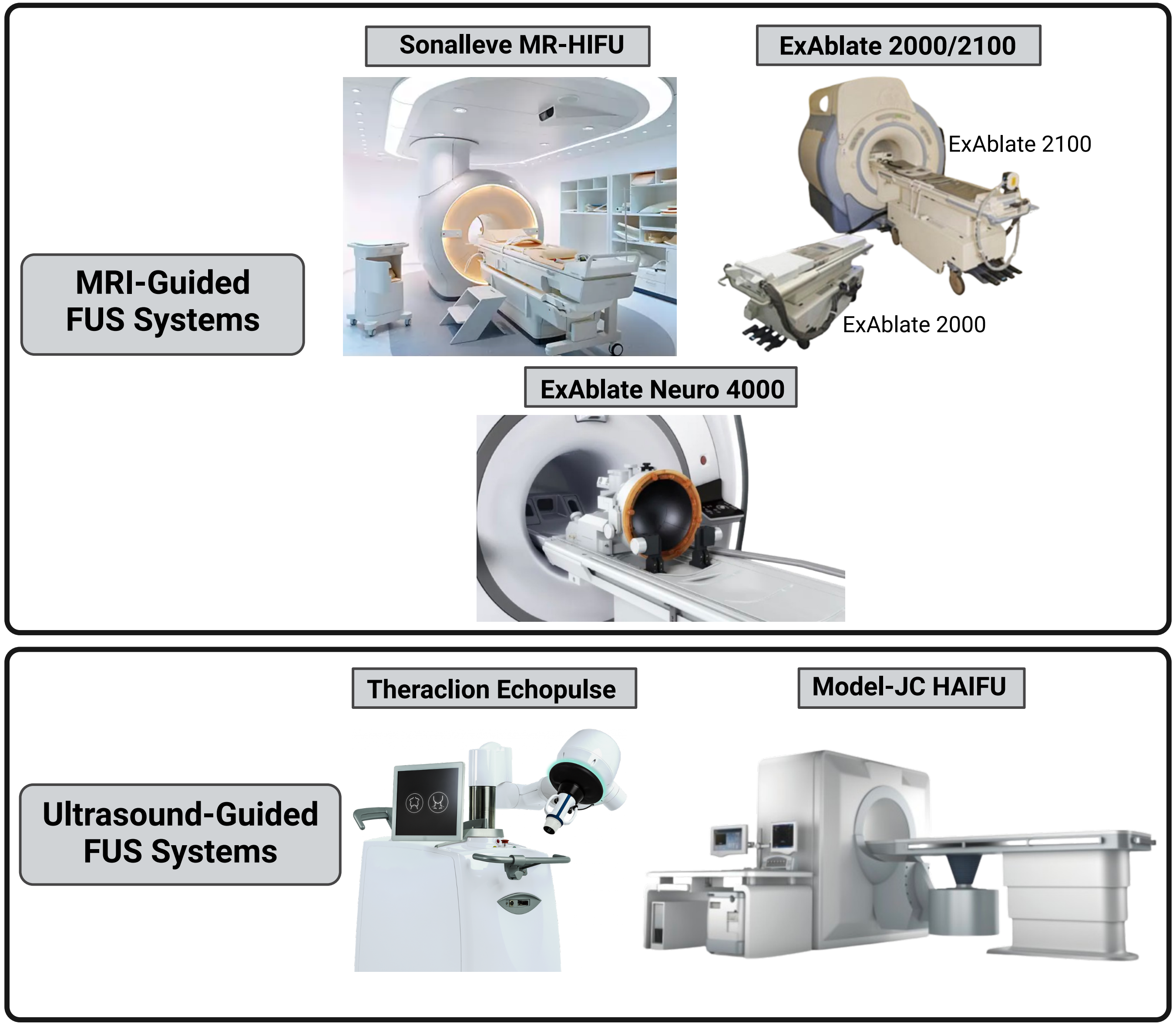

Supplemental Figure 2. Examples of Clinical FUS Devices. Depictions of select imaging-guided FUS systems under past or current use in clinical trials for breast tumor treatment. Not pictured: MUSE MRgFUS System. *Created with Biorender.com.*
