# Supplementary material for: Therapeutic Ultrasound for Multimodal Cancer Treatment: A Spotlight on Breast Cancer
Source: Annu Rev Biomed Eng. Author manuscript; Available in PMC 2025 Sep 5. (PMC12411100; doi:10.1146/annurev-bioeng-103023-111151)
Supplement: Supplemental Material and Tables [file NIHMS2098925-supplement-Supplemental_Material_and_Tables.docx]

Therapeutic Ultrasound for Multimodal Cancer Treatment: A Spotlight on Breast Cancer

Zehra E.F. Demir, B.S.^1^ & Natasha D. Sheybani, Ph.D.^1,2,*^ 

^1^Department of Biomedical Engineering, University of Virginia, Charlottesville, VA

^2^Department of Radiology & Medical Imaging, University of Virginia, Charlottesville, VA

Emails: [qkh9uu@virginia.edu](mailto:qkh9uu@virginia.edu), [nds3sa@virginia.edu](mailto:nds3sa@virginia.edu)

Running Title: Therapeutic Ultrasound for Breast Cancer Treatment

*Corresponding Author:

Natasha D. Sheybani, Ph.D.

Department of Biomedical Engineering

University of Virginia Health System

Box 800759

Charlottesville, VA 22908, USA

Phone: 434-982-4269

Email: nds3sa@virginia.edu

# Combination of FUS with Chemotherapies

## *Free Chemotherapies*

Just as FUS has gained traction as an adjunct to immunotherapies, both preclinical (1–4) and clinical (5) studies have demonstrated the therapeutic impact of combining FUS with conventional chemotherapies. For example, studies have investigated systemic paclitaxel (PTX) administration combined with T-FUS (1–3). One study in MDA-MB-231 and MCF7 BC models demonstrated decreased cell viability and tumor growth control resulting from the combination, *in vitro* and *in vivo*, respectively. Notably, the combination also prolonged overall survival. On examination of PTX pharmacokinetics with and without T-FUS; an increased concentration of PTX was noted at the tumor site within 4 hours of T-FUS exposure (3). Promising observations from numerous preclinical studies have collectively led to initiation of multiple clinical trials combining T-FUS with chemotherapies (i.e. gemcitabine, epirubicin, cyclophosphamide, carboplatin, nab-paclitaxel) and/or immunotherapies (i.e., $\alpha$PD1 and $\alpha$PDL1) in either BC or MBC lesions. Although the results of these clinical trials are pending publication, they underscore the promising direction of combining T-FUS with chemotherapy. One published clinical trial evaluated T-FUS in conjunction with various neoadjuvant chemotherapies (cyclophosphamide, epirubicin and 5-fluorouracil, or docetaxel and cisplatin) in 101 breast cancer patients. There were no major adverse effects noted from T-FUS. The objective response rate for the T-FUS plus neoadjuvant chemotherapy group was higher relative to neoadjuvant chemotherapy alone, resulting in reduced tumor volumes (5).

## *Nanoparticle-Encapsulated Chemotherapies*

The advent of nanomedicine techniques for enhancing efficacy and/or minimizing harsh systemic toxicity of chemotherapies has given way to increasing exploration of NPs (1,4,6–9) and liposomal complexes (2) in the setting of therapeutic ultrasound for BC. Studies deploying various forms of mechanical FUS with PTX-loaded human serum albumin NPs (HSA-NPs) (1), cabazitaxel-encapsuled polymeric NPs (NPMBs) (4), and DOX-loaded albumin NPs (DOX-Al-NPs) have demonstrated efficacy across *in-vitro* (1,4,6) and *in-vivo* (4,6) contexts of MDA-MB-231. The cabazitaxel-encapsuled NPMBs not only inhibited tumor outgrowth, but also led to complete remission of all tumors in a proof-of-concept study. Other formulations, such as the DOX-Al-NPs, have taken advantage of feedback within the TME such as acidity to yield the selective accumulation of DOX around the tumor, facilitating localized intratumoral delivery (6). Similar approaches have been taken in docetaxel (DTX)-loaded liposomes, also known as membrane fusogenic liposomes (MFLs), wherein the capacity of these particles to deliver hydrophobic and hydrophilic substances through fusion of liposomal surfaces can enable reductions in required DTX dose. Results with this platform have demonstrated the ability of repeat FUS-mediated DTX-MFL delivery to reduce BC burden (9).

NP-loaded chemotherapies have also been implemented in combination with SDT (7,8). Indeed, in MDA-MB-231-bearing mice treated with mesoporous silica NPs loaded with DOX and chlorin e6 (MSN-DOX-Ce6) and SDT, significant tumoricidal effects were observed both *in-vitro* and *in-vivo* (7). Another study developed a multifunctional NP system incorporating C46, perfluoropentane, and docetaxel within a PLGA structure (CPDP NPs). When combined with SDT, CPDP NPs not only markedly suppressed 4T1 tumor outgrowth but also mitigated pulmonary metastatic burden in comparison with controls (8).

## *Liposomal Chemotherapies*

Relative to their NP counterparts, liposomes offer distinct advantages in structure and stability and have been similarly leveraged for chemotherapy delivery across BC applications. Compounds such as estrogen-conjugated liposomes encapsulating calcein (here serving as a model drug for doxorubicin; DOX) (10) and hyaluronic acid (HA)-conjugated liposomes housing calcein (11) have been evaluated with mechanical FUS *in-vitro* (1,10), elaborating improved chemotherapeutic delivery. In another *in- vivo* investigation, combination of mechanical FUS with PTX-loaded liposome-MB complexes (PLMC) significantly reduced 4T1 tumor burden and offered improved intratumoral chemotherapy accumulation (1). Temperature-sensitive liposomes (TSLs) have also been studied extensively in combination with FUS hyperthermia, in some cases even being functionalized for improved tumor targeting – as in the example of TSLs encapsulating DOX and featuring tumor-targeting peptide iRGD (12). Magnetic-resonance imaging (MRI)-compatible TSLs have also been developed to allow for real-time image guidance, uptake tracking, and monitoring of drug release within tumors. FUS hyperthermia and systemically administered MR-imageable TSLs encapsulating DOX (iTSL-DOX) resulted in an approximately two-fold increase in contrast agent uptake within triple negative BC xenografts compared to untreated control mice. Enhanced delivery of iTSL-DOX with hyperthermia slowed tumor progression and yielded superlative survival benefit (13). In another example, the co-delivery of carboplatin and SN-38 (irinotecan's super-active metabolite) via iTSLs and FUS hyperthermia offered substantial therapeutic benefit over free drug administration (14).

# References

1. Yan F, Li L, Deng Z, Jin Q, Chen J, Yang W, et al. Paclitaxel-liposome-microbubble complexes as ultrasound-triggered therapeutic drug delivery carriers. J Control Release. 2013 Mar 28;166(3):246–55.

2. Yan F, Li X, Jin Q, Jiang C, Zhang Z, Ling T, et al. Therapeutic ultrasonic microbubbles carrying paclitaxel and LyP-1 peptide: preparation, characterization and application to ultrasound-assisted chemotherapy in breast cancer cells. Ultrasound Med Biol. 2011 May;37(5):768–79.

3. Chen S, Bian H, Duan J. High-Intensity Focused Ultrasound Enhanced Anti-Tumor Activities of Paclitaxel in Breast Cancer in vitro and in vivo. Cancer Manag Res. 2022;14:1303–12.

4. Snipstad S, Berg S, Mørch Ý, Bjørkøy A, Sulheim E, Hansen R, et al. Ultrasound Improves the Delivery and Therapeutic Effect of Nanoparticle-Stabilized Microbubbles in Breast Cancer Xenografts. Ultrasound Med Biol. 2017 Nov;43(11):2651–69.

5. Liu C, Mao XY, Yao F, Jin F. Effects of high-intensity focused ultrasound combined with neoadjuvant chemotherapy treatment on the biological behaviors of breast cancer.

6. Kim D, Lee SS, Yoo WY, Moon H, Cho A, Park SY, et al. Combination Therapy with Doxorubicin-Loaded Reduced Albumin Nanoparticles and Focused Ultrasound in Mouse Breast Cancer Xenografts. Pharmaceuticals (Basel). 2020 Sep 7;13(9):235.

7. Xu P, Yao J, Li Z, Wang M, Zhou L, Zhong G, et al. Therapeutic Effect of Doxorubicin-Chlorin E6-Loaded Mesoporous Silica Nanoparticles Combined with Ultrasound on Triple-Negative Breast Cancer. Int J Nanomedicine. 2020 Apr 21;15:2659–68.

8. Zhang Q, Wang W, Shen H, Tao H, Wu Y, Ma L, et al. Low-Intensity Focused Ultrasound-Augmented Multifunctional Nanoparticles for Integrating Ultrasound Imaging and Synergistic Therapy of Metastatic Breast Cancer. Nanoscale Res Lett. 2021 Apr 29;16(1):73.

9. Kim D, Han J, Park SY, Kim H, Park JH, Lee HJ. Antitumor Efficacy of Focused Ultrasound-MFL Nanoparticles Combination Therapy in Mouse Breast Cancer Xenografts. Materials (Basel). 2020 Mar 2;13(5):1099.

10. Salkho NM, Paul V, Kawak P, Vitor RF, Martins AM, Al Sayah M, et al. Ultrasonically controlled estrone-modified liposomes for estrogen-positive breast cancer therapy. Artif Cells Nanomed Biotechnol. 2018;46(sup2):462–72.

11. Ben Daya SM, Paul V, Awad NS, Al Sawaftah NM, Al Sayah MH, Husseini GA. Targeting Breast Cancer Using Hyaluronic Acid-Conjugated Liposomes Triggered with Ultrasound. J Biomed Nanotechnol. 2021 Jan 1;17(1):90–9.

12. Deng Z, Xiao Y, Pan M, Li F, Duan W, Meng L, et al. Hyperthermia-triggered drug delivery from iRGD-modified temperature-sensitive liposomes enhances the anti-tumor efficacy using high intensity focused ultrasound. J Control Release. 2016 Dec 10;243:333–41.

13. Amrahli M, Centelles M, Cressey P, Prusevicius M, Gedroyc W, Xu XY, et al. MR-labelled liposomes and focused ultrasound for spatiotemporally controlled drug release in triple negative breast cancers in mice. Nanotheranostics. 2021;5(2):125–42.

14. Cressey P, Amrahli M, So PW, Gedroyc W, Wright M, Thanou M. Image-guided thermosensitive liposomes for focused ultrasound enhanced co-delivery of carboplatin and SN-38 against triple negative breast cancer in mice. Biomaterials. 2021 Apr;271:120758.

15. Abe S, Nagata H, Crosby EJ, Inoue Y, Kaneko K, Liu CX, et al. Combination of ultrasound-based mechanical disruption of tumor with immune checkpoint blockade modifies tumor microenvironment and augments systemic antitumor immunity. J Immunother Cancer. 2022 Jan;10(1):e003717.

16. Fite BZ, Wang J, Kare AJ, Ilovitsh A, Chavez M, Ilovitsh T, et al. Immune modulation resulting from MR-guided high intensity focused ultrasound in a model of murine breast cancer. Sci Rep. 2021 Jan 13;11(1):927.

17. Nam GH, Pahk KJ, Jeon S, Park HJ, Kim GB, Oh SJ, et al. Investigation of the Potential Immunological Effects of Boiling Histotripsy for Cancer Treatment. Advanced Therapeutics. 2020;3(8):1900214.

18. Silvestrini MT, Ingham ES, Mahakian LM, Kheirolomoom A, Liu Y, Fite BZ, et al. Priming is key to effective incorporation of image-guided thermal ablation into immunotherapy protocols. JCI Insight. 2017 Mar 23;2(6):e90521.

19. Sheybani ND, Witter AR, Thim EA, Yagita H, Bullock TNJ, Price RJ. Combination of thermally ablative focused ultrasound with gemcitabine controls breast cancer via adaptive immunity. J Immunother Cancer. 2020 Aug;8(2):e001008.

20. Tang R, He H, Lin X, Wu N, Wan L, Chen Q, et al. Novel combination strategy of high intensity focused ultrasound (HIFU) and checkpoint blockade boosted by bioinspired and oxygen-supplied nanoprobe for multimodal imaging-guided cancer therapy. J Immunother Cancer. 2023 Jan;11(1):e006226.

21. Elamir A, Ajith S, Sawaftah NA, Abuwatfa W, Mukhopadhyay D, Paul V, et al. Ultrasound-triggered herceptin liposomes for breast cancer therapy. Sci Rep. 2021 Apr 6;11(1):7545.

22. Li X, Khorsandi S, Wang Y, Santelli J, Huntoon K, Nguyen N, et al. Cancer immunotherapy based on image-guided STING activation by nucleotide nanocomplex-decorated ultrasound microbubbles. Nat Nanotechnol. 2022 Aug;17(8):891–9.

23. Yue W, Chen L, Yu L, Zhou B, Yin H, Ren W, et al. Checkpoint blockade and nanosonosensitizer-augmented noninvasive sonodynamic therapy combination reduces tumour growth and metastases in mice. Nat Commun. 2019 May 2;10(1):2025.

24. Kheirolomoom A, Silvestrini MT, Ingham ES, Mahakian LM, Tam SM, Tumbale SK, et al. Combining activatable nanodelivery with immunotherapy in a murine breast cancer model. J Control Release. 2019 Jun 10;303:42–54.

25. Zhang H, Tang WL, Kheirolomoom A, Fite BZ, Wu B, Lau K, et al. Development of thermosensitive resiquimod-loaded liposomes for enhanced cancer immunotherapy. J Control Release. 2021 Feb 10;330:1080–94.

26. Park EJ, Zhang YZ, Vykhodtseva N, McDannold N. Ultrasound-mediated blood-brain/blood-tumor barrier disruption improves outcomes with trastuzumab in a breast cancer brain metastasis model. J Control Release. 2012 Nov 10;163(3):277–84.

27. Alkins R, Burgess A, Ganguly M, Francia G, Kerbel R, Wels WS, et al. Focused ultrasound delivers targeted immune cells to metastatic brain tumors. Cancer Res. 2013 Mar 15;73(6):1892–9.

28. Kobus T, Zervantonakis IK, Zhang Y, McDannold NJ. Growth inhibition in a brain metastasis model by antibody delivery using focused ultrasound-mediated blood-brain barrier disruption. J Control Release. 2016 Sep 28;238:281–8.

29. Alkins R, Burgess A, Kerbel R, Wels WS, Hynynen K. Early treatment of HER2-amplified brain tumors with targeted NK-92 cells and focused ultrasound improves survival. Neuro Oncol. 2016 Jul;18(7):974–81.

30. O’Reilly MA, Chinnery T, Yee ML, Wu SK, Hynynen K, Kerbel RS, et al. Preliminary Investigation of Focused Ultrasound-Facilitated Drug Delivery for the Treatment of Leptomeningeal Metastases. Sci Rep. 2018 Jun 13;8(1):9013.

**Tables:**

# Supplemental Table 1: Overview of breast cancer subtypes.

| **Hormone Receptor (ER, PR) Expression** | **Breast Cancer Subtype** | | **Immunohistochemistry Signatures** | **Additional Markers** | **Grade** | **Prognosis** | **Therapeutic Intervention** |
| --- | --- | --- | --- | --- | --- | --- | --- |
| HR+ | Luminal A | | ER+, PR+/-, HER2-, Ki67- | N/A | 1\|2 | Good | Hormone Therapy |
|  | Luminal B | | ER+, PR+/-, HER2-, Ki67+ | N/A | 2\|3 | Intermediate | Hormone Therapy |
|  |  |  | ER+, PR+/-, HER2+, Ki67+ | N/A |  | Poor | Hormone Therapy |
| HR- | HER2 over-expression | | ER-, PR-, HER2+, Ki67+ | N/A | 2\|3 | Poor | Trastuzumab, Pertuzumab, Tyrosine Inhibitors |
|  | Triple Negative | Basal-like | ER-, PR-, HER2- | Basal marker (i.e., cytokeratin 5,6,7) | 3 | Poor | Cisplatin, Carboplatin, Paclitaxel, Docetaxel, and Gemcitabine |
|  |  | Claudin-Low | ER-, PR-, HER2- | Cell-to-cell adhesion molecules (i.e., claudin 3,4,7, E-cadherin, & occludin) | 3 | Poor |  |

| **FUS Modality** | | **Reference** | **FUS System** | **FUS Parameters** | **Immunotherapy** | **Model** |  |
| --- | --- | --- | --- | --- | --- | --- | --- |
| **Ablative** | Mechanical | Abe, et al. 2022 (15) | VIFU 2000 System | 1.5 MHz, 2% duty cycle, 5 Hz pulse repetition frequency, 200W, 20 s | αPD-L1 | Syngeneic MM3MG-HER2 model (murine) |  |
|  | Mechanical | Fite, et al. 2021 (16) | MRgFUS Bruker Biospec 7T System, MR-compatible 16-element annular transducer (Imasonic SAS), and MR-compatible transducer position system (Image Guided Therapy) | 3MHz, 0.5% duty cycle, 5ms pulse length, 16.9 MPa, 95.3 MPa (peak positive pressure) | CpG + αPD1 | Bilateral orthotopic n*eu* exon deletion line model (murine) |  |
|  | Boiling Histotripsy | Nam, et al. 2020 (17) | VIFU 2000, Alpinion Medical Sys-  tem | 1.5 MHz, 525W, pulse repetition frequency of 1 Hz, 1% duty  Cycle, and 10ms pulse length | αPD1 | Syngeneic 4T1 model (murine) |  |
|  | Thermal | Silvestrini, et al. 2017 (18) | MRgFUS Bruker Biospec 7T System, MR-compatible 16-element annular transducer (Imasonic SAS), and MR-compatible transducer position system (Image Guided Therapy) | 3MHz, 3.1 MPa, 5W, 65°C | CpG + αPD1 | Bilateral orthotopic n*eu* exon deletion line model (murine) |  |
|  |  |  |  |  |  |  |  |
|  |  |  |  |  |  |  |  |
|  |  | Sheybani, et al. 2020 (19) | (1) Custom in-house single-element USgFUS system and (2) Theraclion Echopulse VTU | (1) 3.3 MHz, 15W, 10s; (2) 3 MHz, 30W, 4s | Gemcitabine + aPD1 | Syngeneic 4T1 model (murine) |  |
|  |  | Fite, et al. 2021 (16) | MRgFUS Bruker Biospec 7T System, MR-compatible 16-element annular transducer (Imasonic SAS), and MR-compatible transducer position system (Image Guided Therapy) | 3MHz, 3.1 MPa, 60°C | CpG + αPD1 | Bilateral orthotopic n*eu* exon deletion line model (murine) |  |
|  |  | Tang, et al. 2023 (20) | JC-200 (Chongqing Haifu Medical Technology) | 120W, 3s | Oxygen-carrying biomimetic perfluorocarbon nanoparticles + αPDL1 | Syngeneic 4T1 model (unilateral murine model) |  |
|  |  |  |  |  |  |  |  |
| **Non-Ablative** | Mechanical | Elamir, et al. 2021 (21) | VCX750 (Sonics & Materials, Inc.) | 20 kHz low-frequency ultrasonic probe, 20s on and 10s off or 20s on and 20s off | pegylated liposomes coated with trastuzumab (TRA-LPs) | SKBR3 & MDA-MB-231 (human BC cell lines) |  |
|  |  | Li, et al. 2022 (22) | Undisclosed FUS system | 1 MHz, 4 W/cm^2^, 60 s, 50% duty cycle, total duration 120s | Nanocomplex-conjugated microbubbles (ncMBs) encapsulating cGAMP + αPD1 | Orthotopic E0771 and 4T1 models (murine) |  |
|  | SDT | Yue, et al. 2019 (23) | Undisclosed FUS system | 1.0 MHz, 1.5 W/cm^2^, 50% duty cycle, 5 min | Liposome-loaded HHME and imiquimod (HHME/R837@Lip) + αPDL1 | Syngeneic 4T1 model (murine) |  |
|  | Hyperthermia | Kheirolomoom, et al. 2019 (24) | Custom 128-element with programmable ultrasound Vantage 256 System | 128 element 1.5 MHz, 2.5 MPa, 100 Hz pulsed repetition frequency, 0-7ms burst duration, 42°C | Temperature-sensitive liposomes (TSL) loaded with a pH-sensitive complex between doxorubicin (Dox) and copper (CuDox) + CpG + αPD1 | Orthotopic n*eu* exon deletion line model (murine) |  |
|  |  | Zhang, et al. 2021 (25) | Custom 128-element with programmable ultrasound Vantage 256 System | 128 element 1.5 MHz, 1.8 MPa, 100 Hz pulsed repetition frequency, 0-8ms burst duration, 42°C | Thermosensitive resiquimod-loaded liposomes + αPD1 | Bilateral orthotopic n*eu* exon deletion line model (murine) |  |
|  | BBB/BTB-O | Park, et al. 2012 (26) | Custom single-element FUS system with MRI feedback | 690 MHz, 0.32W, 0.69 MPa, 10ms burst duration, 1% duty cycle | Trastuzumab | Intracranial inoculation of HER2/neu-positive human breast cancer cells (BT474) (rats) |  |
|  |  | Alkins, et al. 2013 (27) | Custom single-element FUS system with MRI feedback | 551.5 kHz transducer, 0.33 MPa, 10 ms pulses, 1 Hz pulse repetition frequency, 120 s total duration | HER2-targeted NK-92 cells | Intracranial inoculation of HER2-amplfied MDA-MB-231 cells (rats) |  |
|  |  | Kobus, et al. 2016 (28) | Custom single-element FUS system with MRI feedback | 690 MHz, 10ms burst duration, 1 Hz repetition frequency, 0.4 W or 0.7 W, 0.46 MPa or 0.62 MPa | Trastuzumab and pertuzumab | Intracranial inoculation of MDA-MB-361- HER2 cells (rats) |  |
|  |  | Alkins, et al. 2016 (29) | Custom single-element FUS system with MRI feedback | 551.5 kHz transducer, 10 ms pulses, 2 Hz pulse repetition frequency, 120 s total duration | HER2- targeted NK-92 cells | Intracranial inoculation of HER2-amplfied MDA-MB-231 cells (rats) |  |
|  |  | O’Reilly, et al. 2018 (30) | RK-100  (FUS Instruments, Inc.) | 551.5 kHz, 10 ms burst delivered at a 1 Hz pulse repetition frequency for 2 minutes, 0.25 MPa | Trastuzumab | Leptomeningeal metastases induced via injection of MDA-MB-231-H2N cells into subarachnoid space of thoracic spine (rats) |  |

**Supplemental Table 2**: Overview of pre-clinical rodent studies combining FUS modalities with immunotherapy agent alone, combined with other agents, and/or in NP formulation for treatment of breast cancer.
